# Post-Exposure 3-Methyladenine Is Associated with Lower Indices of Hepatic Injury and Fibrotic Remodeling After Chronic Sodium Arsenite Exposure in Mice

**DOI:** 10.3390/toxics14100873

**Published:** 2026-09-30

**Authors:** Yu Hui, Qin Liu

**Affiliations:** 1 School of Public Health, Chongqing Medical University, Chongqing 401331, China; huiyu@xjsmc.edu.cn; 2 School of Public Health, Xinjiang Second Medical College, Karamay 834000, China

**Keywords:** sodium arsenite, 3-methyladenine, post-exposure intervention, hepatic injury, liver fibrosis, autophagy-related proteins

## Abstract

This study aimed to evaluate 3-methyladenine (3-MA) as a post-exposure intervention for arsenic-associated hepatic injury and fibrotic remodeling, motivated by its reported antifibrotic effects. Female C57BL/6J mice received sodium arsenite via drinking water for 20 weeks at a nominal target dose of 1.08 mg/kg body weight/day, based on our group’s previous experiments. The remaining exposed mice were randomized to intraperitoneal vehicle (*n* = 8) or 3-MA (*n* = 9; 5 μg/g body weight), administered twice weekly for 4 weeks after arsenic withdrawal. The 3-MA dose and frequency were based on a published carbon tetrachloride-induced fibrosis study. Serum markers, liver histology, and hepatic proteins were assessed. Serum alanine aminotransferase (ALT) and aspartate aminotransferase (AST) trajectories differed between groups, with lower Day-28 activities after 3-MA; the ALT difference persisted after baseline adjustment (*p* = 0.0011). Day-28 serum hyaluronic acid, N-terminal propeptide of type III procollagen, tumor necrosis factor-α, collagen volume fraction, and α-smooth muscle actin immunoreactivity were also lower than with vehicle. These differences were accompanied by changes in nuclear factor-kappa B- and autophagy-related proteins. Post-exposure 3-MA was associated with lower hepatic injury and fibrotic remodeling indices, but an autophagy-dependent mechanism and within-animal fibrosis regression were not established.

## 1. Introduction

Liver fibrosis develops as a dynamic response to chronic injury. Activated hepatic stellate cells acquire a myofibroblast-like phenotype, express α-smooth muscle actin (α-SMA), and deposit extracellular matrix [1,2,3]. Fibrosis may regress after removal of the underlying insult, although the extent and timing of recovery depend on injury severity, persistent inflammation, stellate-cell fate, and extracellular-matrix remodeling [2,4,5,6]. The early period after exposure withdrawal may therefore represent a distinct window for intervention.

Inorganic arsenic remains a widespread drinking-water contaminant and a major public-health concern [7,8,9]. The liver is an important site of arsenic biotransformation [8]. Experimental sodium arsenite exposure has been associated with hepatic injury, oxidative stress, inflammation, and fibrotic remodeling [10,11,12,13,14]. Experimental studies link sodium arsenite-induced hepatic injury to impaired antioxidant defenses and increased lipid peroxidation [10]. Arsenic-induced redox imbalance can also promote signaling between hepatocytes and hepatic stellate cells. A recent study showed that disrupted glutathione metabolism and NLRP3 inflammasome activation in arsenic-exposed hepatocytes were involved in stellate-cell activation and liver fibrosis [13]. Extracellular-matrix remodeling may further reinforce this fibrogenic response. Increased matrix stiffness promotes fibrogenic changes in hepatic stellate cells through AP-1-associated changes in chromatin accessibility [15]. Together, these observations suggest that oxidative injury, inflammatory signaling, and matrix-derived mechanical cues can interact during fibrogenesis. Previous work has examined hepatoprotective treatment during ongoing sodium arsenite exposure [10]. Whether hepatic injury and fibrotic remodeling remain modifiable after exposure withdrawal requires further investigation.

Inflammation links hepatocellular injury to fibrogenesis. Nuclear factor-kappa B (NF-κB)-dependent signaling regulates cytokine production, immune-cell recruitment, cell survival, and tissue remodeling [16,17,18]. Autophagy contributes to hepatic homeostasis but has cell-type- and stage-dependent effects in liver injury and fibrosis [19,20,21,22,23]. Autophagy interacts with inflammatory signaling [21,24]. Experimental studies using sodium arsenite or arsenic trioxide have reported changes in autophagy-related markers or autophagy-inflammasome signaling in hepatic models [14,25,26]. These observations motivated the assessment of NF-κB, LC3, and p62 as molecular correlates in the present study. However, static protein measurements cannot establish autophagic flux or define a causal signaling sequence [27].

3-Methyladenine (3-MA) is a pharmacological inhibitor of class I and class III phosphoinositide 3-kinases (PI3Ks). Its effects on autophagy depend on treatment duration and nutrient availability, limiting its use as a selective mechanistic probe [28]. In a carbon tetrachloride-induced mouse model of liver fibrosis, 3-MA reduced collagen deposition and hepatic stellate-cell activation, accompanied by changes in NF-κB- and autophagy-related proteins [29]. These findings provided a rationale for evaluating 3-MA after arsenic withdrawal. However, evidence from a different injury model does not establish its efficacy or mechanism in this post-exposure setting. We hypothesized that 3-MA administration after arsenic withdrawal would be associated with lower indices of hepatic injury and fibrotic remodeling than vehicle administration. This study aimed to evaluate these post-exposure hepatic outcomes in female mice following chronic sodium arsenite exposure. A further aim was to examine whether differences in hepatic outcomes were accompanied by changes in NF-κB- and autophagy-related protein markers.

## 2. Materials and Methods

### 2.1. Animals and Experimental Design

Thirty-two specific pathogen-free female C57BL/6J mice, aged 6 weeks and weighing 18–22 g, were purchased from Hubei Zhishu Zhihui Biotechnology Co., Ltd. (Wuhan, China). Previous arsenic studies in our laboratory had focused on male mice. Female mice were therefore selected for the present study to examine hepatic responses and post-exposure intervention effects in females. Estrous cycles were not monitored. The mice were housed in the Experimental Animal Center of Xinjiang Medical University at 20–25 °C and 50–60% relative humidity under a 12 h light/dark cycle. Standard chow (Jiangsu Xietong Pharmaceutical Bio-Engineering Co., Ltd., Nanjing, China) and water were provided ad libitum. After a 1-week acclimatization period, the mice were allocated using a random-number table to an unexposed Control cohort (*n* = 6) or a sodium arsenite-exposed cohort (*n* = 26). At the start of the exposure phase, Control mice were housed in two cages of three mice each. At week 8, all three Control mice from one cage underwent terminal sampling, while the three mice in the other cage remained in their original cage until terminal sampling at week 20. Arsenic-exposed mice were initially housed at four to five per cage. At the start of the intervention phase, mice were housed at four to five per cage. Arsenic-exposed mice were not regrouped after scheduled terminal sampling. No formal a priori sample size calculation was performed. Animal numbers were informed by a previous study [14] and increased to allow for potential animal loss during the experiment.

Sodium arsenite (NaAsO_2_; CAS No. 7784-46-5; catalog No. W12590; purity ≥ 90%) was purchased from Shanghai Yuanye Biotechnology Co., Ltd. (Shanghai, China). Sodium arsenite was dissolved in autoclaved reverse-osmosis (RO) water to prepare the arsenic-containing drinking water, which was provided ad libitum to the exposed cohort for 20 weeks. The drinking-water route was selected to model chronic oral exposure through contaminated water, allowing arsenite intake during normal drinking behavior rather than delivery as discrete bolus doses by gavage. The arsenic-containing drinking water was not acidified. The nominal target dose was 1.08 mg/kg body weight/day, selected on the basis of previous experiments conducted by our research group. No preliminary dose-ranging experiments were performed as part of the present study. Cage-level water consumption was determined from changes in bottle mass and normalized to the number of mice and the duration of each measurement interval. Water mass was converted to volume assuming a density of 1 g/mL. At each water-replacement interval, typically every 2 days, the working NaAsO_2_ concentration was recalculated using the corresponding weekly group mean body weight and cage-derived mean daily water intake per mouse:NaAsO_2_ concentration (mg/L) = nominal target dose (mg/kg body weight/day) × group mean body weight (kg)/mean daily water intake per mouse (L/day).The calculated working concentrations ranged from 3.10 to 22.68 mg/L, with a median of 6.77 mg/L. Control mice received autoclaved RO water without sodium arsenite. Body weight and general condition were assessed weekly throughout the exposure period.

To characterize model progression, mice were sampled at Weeks 8, 16, and 20 during arsenic exposure. Three Control and three exposed mice were sampled at Week 8, three additional exposed mice at Week 16, and three Control and three exposed mice at Week 20. These animals did not enter the intervention phase. All sampled mice were fasted overnight before blood and liver collection.

Following the week 20 assessment, the remaining arsenic-exposed mice (*n* = 17) were randomly assigned to the Withdrawal + Vehicle group (n = 8) or Withdrawal + 3-MA group (*n* = 9). Blood obtained after an overnight fast immediately before the intervention served as the Day 0 pretreatment baseline. Serum measurements from Control mice sampled at week 20 served only as descriptive references and were excluded from intervention-phase statistical comparisons. Arsenic exposure was then discontinued, and all remaining mice received autoclaved RO water. 3-Methyladenine (3-MA; CAS No. 5142-23-4; catalog No. HY-19312; purity 99.91%) was purchased from MedChemExpress (MCE, USA). The 3-MA solution was freshly prepared in sterile normal saline before each administration. Mice in the Withdrawal + Vehicle group received an equivalent volume of normal saline. Mice in the Withdrawal + 3-MA group received 3-MA at 5 µg/g body weight by intraperitoneal injection. Both treatments were administered twice weekly for 4 weeks. The 3-MA dose and dosing frequency were selected based on a previous study of carbon tetrachloride-induced liver fibrosis [29]. The 4-week post-withdrawal intervention period was prespecified in the study design and was not based on a specific published protocol or pilot experiment. No preliminary dose-ranging experiments were performed for 3-MA in the present study. Blood samples were collected from the retro-orbital sinus using non-heparinized microhematocrit capillaries at Day 0, Week 2, and Week 4 under topical local anesthesia without general anesthesia. One drop of 0.5% tetracaine ophthalmic solution was instilled into the eye selected for sampling 1 min before each collection. The blood volume collected at each sampling was not recorded. Immediately after blood collection, gentle pressure was applied to the sampled eyelid with sterile dry gauze until bleeding stopped. After hemostasis, the mice were returned to their home cages. They were checked once within 24 h for persistent bleeding, proptosis, corneal clouding, or abnormal eyelid closure. No overt abnormalities were noted during these post-collection observations.

During the 4-week intervention period, the general health of the mice was checked every 3 days. Withdrawal criteria included severe emaciation, a hunched posture, and a rough, unkempt coat. Affected animals were to be withdrawn from further intervention and observed for improvement, without re-entering the study. No animals were withdrawn from the study or excluded from the final analysis.

After the final blood collection, mice were anesthetized by intraperitoneal injection of 2,2,2-tribromoethanol at 250 mg/kg body weight. The anesthetic was obtained from Sigma-Aldrich (catalog No. T48402-100G; purity 97%; St. Louis, MO, USA). Under deep anesthesia, the mice were euthanized by cervical dislocation, and liver tissues were harvested.

A portion of each liver was fixed in 4% paraformaldehyde for histological and immunohistochemical analyses. The remaining liver tissue was snap-frozen and stored at −80 °C for protein analysis. Histological and immunohistochemical quantification was performed by investigators blinded to group allocation. 

All procedures were approved by the Animal Ethics Committee of Xinjiang Medical University (approval No.IACUC-20220728-09). The experiments were conducted in accordance with institutional guidelines for laboratory-animal care and reported in accordance with the ARRIVE guidelines.

**Figure 1:**
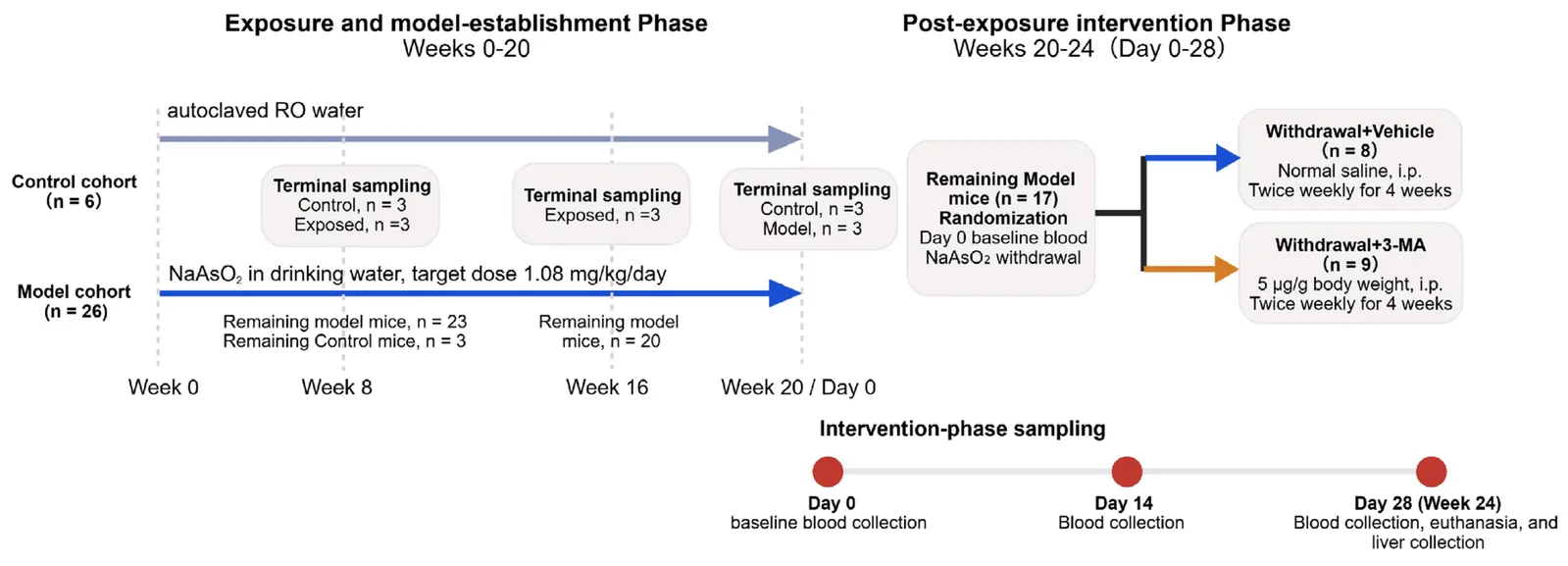
Experimental design of chronic sodium arsenite exposure and post-exposure intervention with 3-methyladenine. The Control cohort initially comprised six mice receiving autoclaved reverse-osmosis (RO) water, whereas the Model cohort comprised 26 mice receiving sodium arsenite (NaAsO_2_) in drinking water at a target dose of 1.08 mg/kg body weight/day during the 20-week exposure phase. At Week 8, three Control mice and three Model mice underwent terminal sampling. At Week 16, only three Model mice underwent terminal sampling; no Control mice were sampled. At Week 20, three Control mice and three Model mice underwent terminal sampling. Following terminal sampling at Week 20, the remaining Model mice were randomly assigned to the Withdrawal + Vehicle group (*n* = 8) or the Withdrawal + 3-MA group (*n* = 9). Baseline blood was collected on Day 0 before arsenic withdrawal and the first injection. During the subsequent 4-week intervention, both groups received autoclaved RO water. Normal saline or 3-methyladenine (3-MA; 5 μg/g body weight) was administered intraperitoneally twice weekly. Blood samples were collected on Days 0, 14, and 28, and liver tissues were collected after euthanasia on Day 28.

Figure 1 outlines the experimental design, including sodium arsenite exposure, terminal sampling at Weeks 8, 16, and 20, and the post-exposure intervention phase.

### 2.2. Serum Biochemical and Fibrosis-Related Marker Assays

Blood samples were centrifuged at 3500 rpm for 10 min. The serum was collected and maintained at 4 °C pending analysis. Serum alanine aminotransferase (ALT) and aspartate aminotransferase (AST) activities were determined using kinetic assay kits from Wuhan Kebai Biotechnology Co., Ltd. (Wuhan, China). ALT activity was measured using the International Federation of Clinical Chemistry and Laboratory Medicine (IFCC)-recommended method. The primary and secondary wavelengths were 340 and 405 nm, respectively. AST activity was measured using a kinetic aspartate–substrate method at 340 nm. Absorbance changes for both assays were recorded from 60 to 240 s. Measurements were performed using a BS-360S automated chemistry analyzer (Shenzhen Mindray Bio-Medical Electronics Co., Ltd., Shenzhen, China). ALT and AST activities were calculated according to the manufacturer’s instructions and expressed in U/L. For ALT and AST assays, samples with insufficient volume were diluted threefold before measurement, and enzyme activities were corrected for the dilution factor.

Serum fibrosis-related markers and tumor necrosis factor-α (TNF-α) were quantified using mouse-specific enzyme-linked immunosorbent assay (ELISA) kits. The kits measured TNF-α (catalog No. ABE-M4406), hyaluronic acid (HA; ABE-M3906), and the N-terminal propeptide of type III procollagen (PIIINP; ABE-M4203). Additional kits measured type IV collagen (COL4; ABE-M3689) and laminin (LN; ABE-M4067). All ELISA kits were obtained from Wuhan ABcobio Biological Engineering Co., Ltd. (Wuhan, China). The assay ranges were 4.69~300 pg/mL for TNF-α, 0.16~10 ng/mL for PIIINP, and 1.56~100 ng/mL for HA, COL4, and LN. Assays were performed according to the manufacturer’s instructions. TNF-α, COL4, LN, and PIIINP were measured using sandwich ELISAs, whereas HA was measured using a competitive ELISA. For the sandwich assays, 100 μL of each standard or sample was incubated in the coated wells at 37 °C for 90 min. The liquid was discarded without washing, and 100 μL of the biotinylated antibody working solution was added, followed by incubation at 37 °C for 1 h. For the HA assay, 50 μL of each standard or sample was mixed with 50 μL of the biotinylated antibody working solution and incubated at 37 °C for 45 min. For all five assays, the wells were then washed three times and incubated with 100 μL of the horseradish peroxidase conjugate working solution at 37 °C for 30 min. After five washes, 90 μL of tetramethylbenzidine substrate was added, and the plates were incubated at 37 °C for 15–20 min protected from light. Reactions were stopped with 50 μL of stop solution per well. Incubation steps were performed using an ICV-450 constant-temperature incubator (AS ONE, Osaka, Japan). Absorbance was measured at 450 nm within 5 min after stopping the reaction using a Multiskan MK3 microplate reader (Thermo Scientific, Waltham, MA, USA). Analyte concentrations were calculated from the corresponding standard curves.

At the week 20 model-validation endpoint, serum ALT and AST activities and serum HA, PIIINP, LN, and COL4 concentrations were measured. During the intervention period, ALT and AST activities were assessed at Day 0, week 2, and week 4 to monitor hepatocellular injury. Serum HA, PIIINP, and TNF-α concentrations were measured at the end of the 4-week intervention.

### 2.3. Histological and Immunohistochemical Analyses

At the model-validation endpoint (Week 20) and the end of the 4-week intervention, liver samples were fixed in 4% paraformaldehyde. Fixed tissues were dehydrated through graded ethanol, cleared in xylene, embedded in paraffin, and sectioned at a thickness of 4 μm. Sections were subjected to hematoxylin and eosin (H&E) staining, Masson’s trichrome staining, and α-smooth muscle actin (α-SMA) immunohistochemistry. Histological assessment and image quantification were performed by investigators blinded to group allocation.

#### 2.3.1. Hematoxylin and Eosin Staining

Paraffin-embedded liver sections were deparaffinized in xylene and rehydrated through a graded ethanol series. The sections were stained with hematoxylin, differentiated, blued, and counterstained with eosin. They were then dehydrated, cleared, and mounted with neutral resin mounting medium. Hepatic morphology was examined under a light microscope (Nikon, Tokyo, Japan). H&E findings were assessed qualitatively, without a histological injury score.

#### 2.3.2. Masson’s Trichrome Staining

Sections were deparaffinized, rehydrated, and stained using a Modified Masson’s Trichrome Stain Kit (catalog no. G1346; Beijing Solarbio Science & Technology Co., Ltd., Beijing, China). Staining was performed according to the manufacturer’s instructions. After dehydration, clearing, and mounting, collagen deposition was examined by light microscopy. Whole-slide images were acquired using a PANNORAMIC whole-slide scanner (3DHISTECH, Budapest, Hungary) and viewed using SlideViewer 2.5 (3DHISTECH). Three randomly selected, non-overlapping fields per mouse were analyzed at 400× magnification using Image-Pro Plus 6.0 (Media Cybernetics, Rockville, MD, USA). Collagen volume fraction (CVF) was calculated as follows:

$$\mathrm{CVF}\;(\%)\;=\frac{\mathrm{collagen}-\mathrm{positive}\;\mathrm{pixel}\;\mathrm{area}}{\mathrm{total}\;\mathrm{tissue}\;\mathrm{pixel}\;\mathrm{area}}\times 100$$

The values obtained from the three fields were averaged to generate one biological value for each mouse.

#### 2.3.3. Immunohistochemical Staining and Quantification of α-SMA

Sections were deparaffinized, rehydrated, and subjected to heat-induced antigen retrieval in citrate buffer. Endogenous peroxidase activity was blocked with 3% hydrogen peroxide. Nonspecific binding was subsequently blocked with 10% serum. The sections were incubated overnight at 4 °C with a mouse monoclonal anti-α-SMA/ACTA2 antibody (clone 1A4; 1:5000; catalog no. BM0002; Boster Biological Technology, Wuhan, China; RRID: AB_2811044). After washing, immunoreactive signals were detected using an HRP-conjugated anti-mouse IgG Super Vision Assay Kit (catalog no. SV0001; Boster Biological Technology, Wuhan, China). Detection was performed according to the manufacturer’s instructions. Immunoreactivity was visualized using 3,3′-diaminobenzidine (DAB), followed by hematoxylin counterstaining. α-SMA immunoreactivity was quantified using Saiviewer 2.3.1 (Servicebio, Wuhan, China). Cells were classified as negative, weakly stained, moderately stained, or strongly stained. The corresponding intensity scores were 0, 1, 2, and 3, respectively. The H-score was calculated as follows:

H-score = (percentage of weakly stained cells × 1) + (percentage of moderately stained cells × 2) + (percentage of strongly stained cells × 3)

The resulting H-score ranged from 0 to 300, with higher values indicating greater overall α-SMA immunoreactivity. Three randomly selected, non-overlapping regions of interest were analyzed per mouse. The three H-scores were averaged to generate one biological value for each mouse.

### 2.4. Western Blot Analysis

Frozen liver tissues collected at the intervention endpoint were analyzed by Western blotting. All mice in the Withdrawal + Vehicle (*n* = 8) and Withdrawal + 3-MA (*n* = 9) groups were included. Total protein was extracted using RIPA lysis buffer supplemented with protease and phosphatase inhibitors (Wuhan ABcobio Biological Engineering Co., Ltd., Wuhan, China). Protein concentrations were determined using a bicinchoninic acid assay.

Equal amounts of protein (20 μg per lane) were separated by sodium dodecyl sulfate–polyacrylamide gel electrophoresis and transferred onto polyvinylidene difluoride membranes at 300 mA. A 12% resolving gel and a transfer time of 20 min were used for LC3B. The other proteins were separated using 10% gels and transferred for 30 min.

Membranes were blocked with 3% bovine serum albumin for 30 min and incubated at 4 °C for approximately 15 h with antibodies against phospho-NF-κB p65 (Ser536) (1:1000; catalog no. 3033; Cell Signaling Technology, Danvers, MA, USA), total NF-κB p65/RELA (1:1000; A00284-1; Boster Biological Technology, Wuhan, China), IκBα (1:1000; MA01139; Boster), LC3B (1:1000; BM4827; Boster), p62/SQSTM1 (1:1000; 5114; Cell Signaling Technology), or GAPDH (1:10,000; 60004-1-Ig; Proteintech, Wuhan, China). Membranes were subsequently incubated for 30 min with the appropriate HRP-conjugated goat anti-rabbit or goat anti-mouse IgG secondary antibody (1:5000; catalog nos. 074-1506 and 074-1806; Kirkegaard & Perry Laboratories, Gaithersburg, MD, USA).

Bands were visualized using enhanced chemiluminescence and captured with a ChemiScope 6100 imaging system (Clinx Science Instruments, Shanghai, China). Band intensities were quantified using AlphaEaseFC version 3.1.2 (Alpha Innotech, San Leandro, CA, USA). The p-p65/total p65 ratio was calculated as an NF-κB-related phosphorylation index. IκBα and p62 were normalized to GAPDH, whereas LC3B processing was quantified using the LC3B-II/LC3B-I band-intensity ratio. Densitometric analyses included all mice, although only three representative lanes per group are displayed in the figures. Uncropped Western blot images are provided in Supplementary File S1.

### 2.5. Statistical Analysis

Statistical analyses were performed using SPSS version 26.0 and R 4.6.1. Normality was assessed with the Shapiro–Wilk test. Data are presented as mean ± SD or median (interquartile range), as appropriate. The mouse was the statistical unit; for CVF and α-SMA H-score, measurements from three fields or regions were averaged to obtain one value per mouse. Independent two-group comparisons used an unpaired Student’s t-test or Mann–Whitney U test, as appropriate. ALT and AST were analyzed separately using two-way mixed-design repeated-measures ANOVA (group × time). Sphericity was assessed using Mauchly’s test, with Greenhouse–Geisser correction when required. Sidak correction was applied across the three between-group comparisons per marker (Days 0, 14, and 28). Post hoc sensitivity analyses included Day-28 ALT ANCOVA adjusted for Day-0 ALT and an unpaired t-test comparing AST changes from Day 0 to Day 28. Baseline-by-group interactions were examined to assess the common-slope assumption for ANCOVA. All tests were two-sided, with *p* < 0.05 considered statistically significant. Numerical source data are provided in Supplementary Data S1.

## 3. Results

### 3.1. Week-20 Sampling Shows Hepatic Injury and Fibrotic Remodeling After Chronic Sodium Arsenite Exposure

At Week 20, three Control and three Model mice underwent biochemical and histological analyses. The remaining Model mice entered the intervention phase.

**Figure 2:**
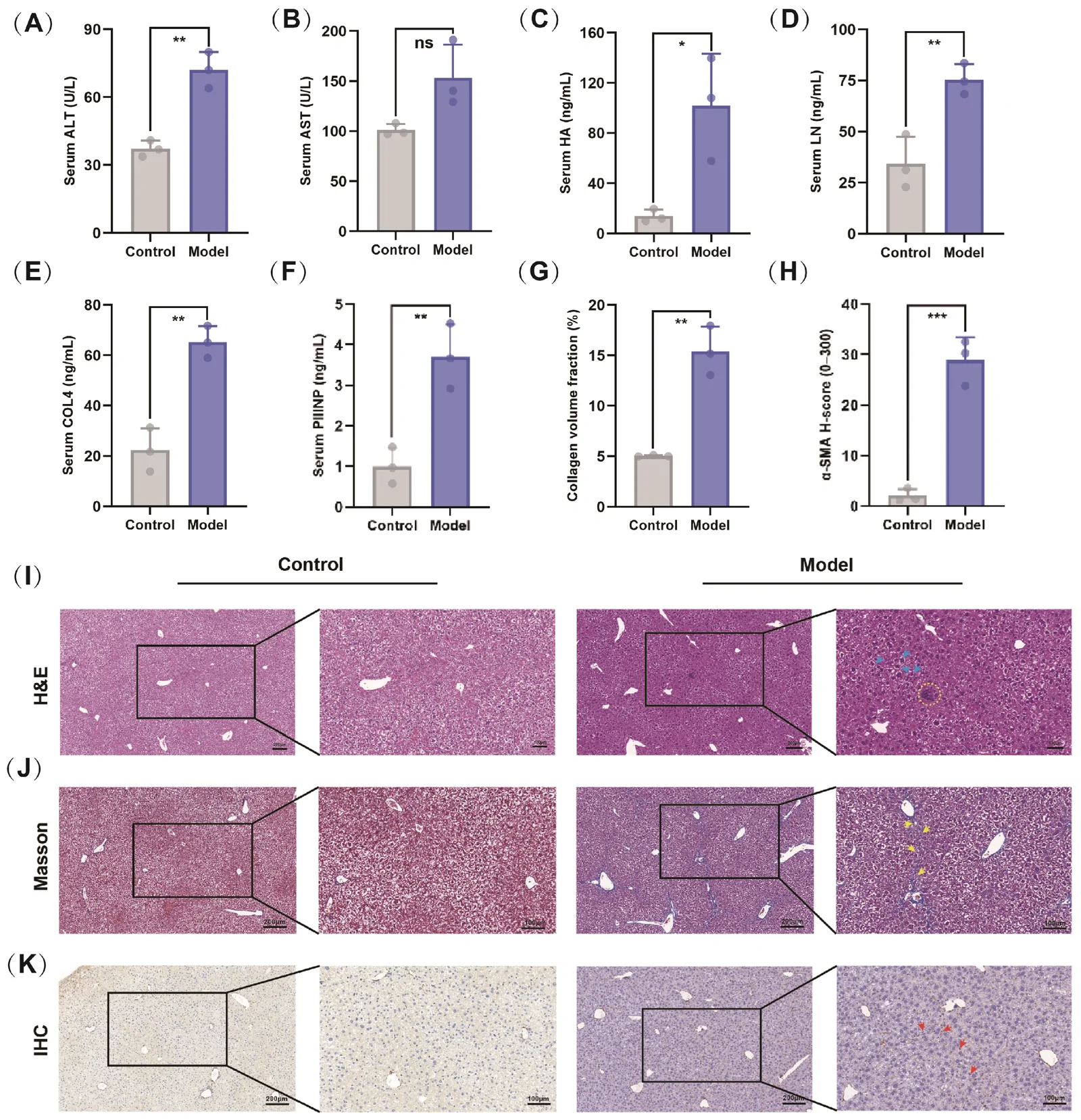
Hepatic injury and fibrotic remodeling after 20 weeks of sodium arsenite exposure. (**A**,**B**) Serum alanine aminotransferase (ALT; (**A**)) and aspartate aminotransferase (AST; (**B**)) activities. (**C**–**F**) Serum concentrations of hyaluronic acid (HA; (**C**)), laminin (LN; (**D**)), type IV collagen (COL4; (**E**)), and procollagen type III N-terminal propeptide (PIIINP; (**F**)). (**G**) Collagen volume fraction determined from Masson’s trichrome-stained sections. (**H**) α-Smooth muscle actin (α-SMA) H-score. (**I**–**K**) Representative hematoxylin and eosin (H&E) staining (**I**), Masson’s trichrome staining (**J**), and α-SMA immunohistochemistry (**K**). Boxed regions are shown at higher magnification. Blue arrows indicate altered hepatocyte morphology, yellow dashed circles indicate focal cellular aggregates, yellow arrows indicate collagen deposition and fibrous cords, and red arrows indicate α-SMA-positive cells. Scale bars = 200 μm in the low-magnification images and 100 μm in the enlarged images. Dots represent individual mice, and bars show mean ± SD; *n* = 3 mice per group for panels (**A**–**H**). Statistical comparisons were performed as described in the Materials and methods. * *p* < 0.05, ** *p* < 0.01, and *** *p* < 0.001; ns, not significant.

Serum ALT was higher in the Model group than in the Control group at Week 20 (*p* < 0.01; Figure 2A). Serum AST was numerically higher in Model mice, but the difference was not statistically significant (Figure 2B). Model mice also had higher serum HA, LN, COL4, and PIIINP concentrations (Figure 2C–F).

Representative H&E-stained sections showed altered hepatocyte morphology and focal cellular aggregates in Model mice (Figure 2I). Masson’s trichrome staining showed increased collagen deposition and local fibrous cords (Figure 2J), and collagen volume fraction was correspondingly higher (Figure 2G). Model livers also showed greater α-SMA immunoreactivity (Figure 2K) and a higher α-SMA H-score (Figure 2H).

These biochemical and histological findings characterized hepatic injury and fibrotic remodeling in the sampled Model mice. Because the Week-20 comparison included only three mice per group, it was used to characterize the pre-intervention phenotype.

### 3.2. Serum Aminotransferase Trajectories and Hepatic Histology After Arsenic Withdrawal

After arsenic withdrawal, mice in the Withdrawal + Vehicle (n = 8) and Withdrawal + 3-MA (n = 9) groups were followed for 28 days. Serum ALT and AST were measured on Days 0, 14, and 28. Week-20 Control values served only as a descriptive reference and were excluded from intervention-phase comparisons.

**Figure 3:**
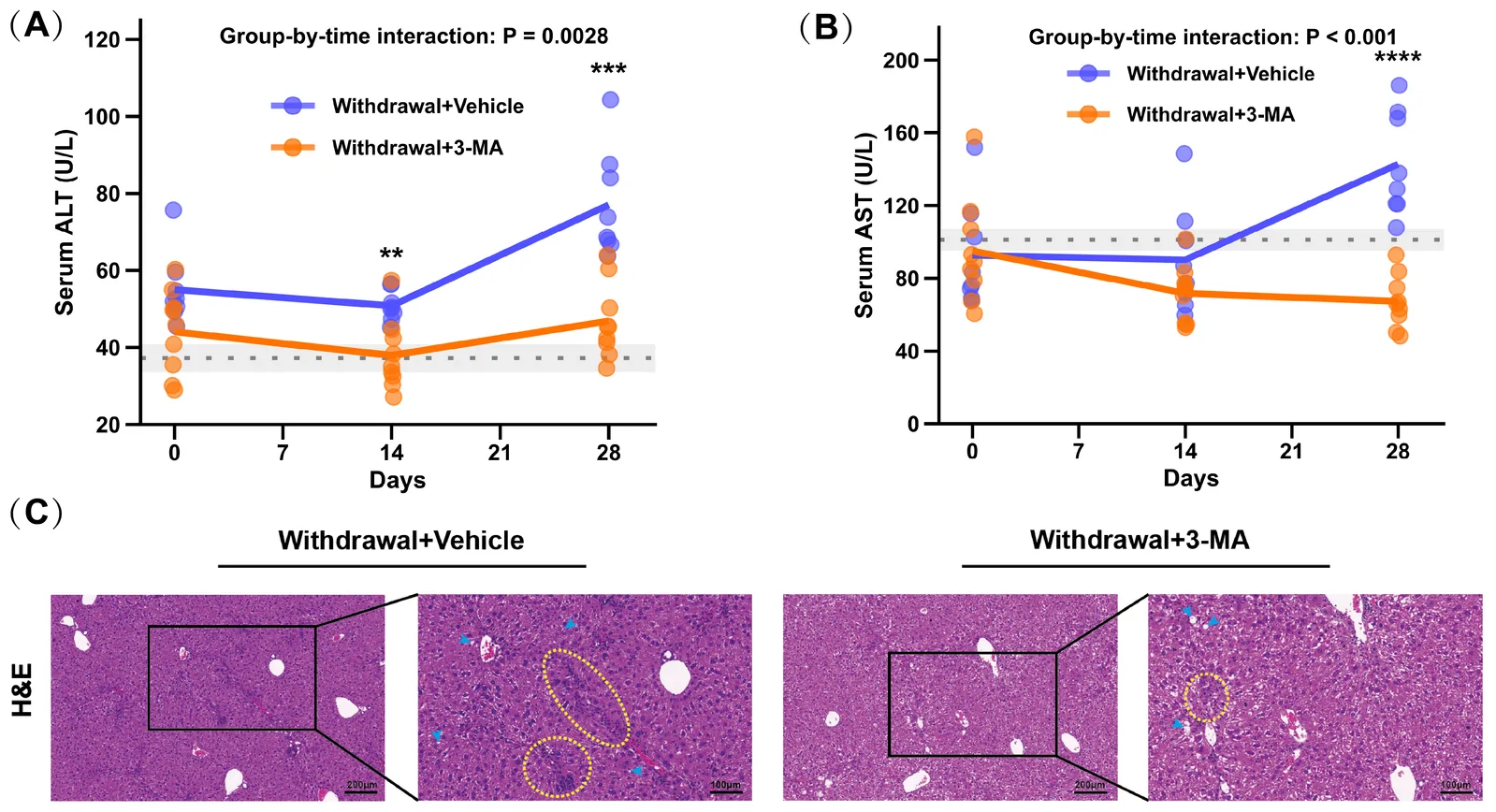
Serum aminotransferase trajectories and representative hepatic histology during post-exposure 3-MA administration. (**A**,**B**) Serum ALT (**A**) and AST (**B**) on Days 0, 14, and 28 after arsenic withdrawal (Withdrawal + Vehicle, *n* = 8; Withdrawal + 3-MA, *n* = 9). Dots represent individual mice; solid lines connect group means. The gray dotted line and band show the Week-20 Control mean ± SD (*n* = 3) as a descriptive reference, excluded from inferential comparisons. Time-by-group interactions were tested using two-way mixed-design repeated-measures ANOVA. Stars indicate between-group comparisons at each time point, with Sidak correction across three comparisons per marker. (**C**) Representative Day-28 H&E sections; boxed regions are enlarged. Blue arrows indicate altered hepatocyte morphology, and yellow dashed circles indicate focal cellular aggregates. Scale bars: 200 μm in overview images and 100 μm in enlarged images. ** *p* < 0.01, *** *p* < 0.001, and **** *p* < 0.0001.

ALT trajectories differed between groups (time-by-group interaction: *F*(2,30) = 7.172, *p* = 0.0028, partial *η*^2^ = 0.323; Figure 3A). At Day 0, ALT was numerically lower in the Withdrawal + 3-MA group than in the Withdrawal + Vehicle group (44.04 ± 10.96 versus 54.99 ± 9.31 U/L; Sidak-adjusted *p* = 0.1250). ALT was lower in the Withdrawal + 3-MA group at Days 14 and 28 (adjusted *p* = 0.0070 and *p* = 0.0003, respectively). In the post hoc ANCOVA, the Day-28 difference remained after adjustment for Day-0 ALT (Withdrawal + 3-MA minus Withdrawal + Vehicle: −27.49 U/L; 95% CI, −41.92 to −13.07; *p* = 0.0011).

AST trajectories also differed between groups (time-by-group interaction: *F*(2,30) = 27.298, *p* < 0.001, partial *η*^2^ = 0.645; Figure 3B). Between-group differences were not significant at Days 0 and 14 (Sidak-adjusted *p* = 0.9975 and *p* = 0.3184, respectively), whereas AST was lower in the Withdrawal + 3-MA group at Day 28 (adjusted *p* < 0.0001). A baseline-by-group interaction was detected in the Day-28 AST ANCOVA model (*p* = 0.0320), so a common-slope adjusted group difference was not reported. The post hoc comparison of Day-28 minus Day-0 changes showed a lower change in the Withdrawal + 3-MA group (Withdrawal + 3-MA minus Withdrawal + Vehicle: −78.05 U/L; 95% CI, −105.69 to −50.40; *p* < 0.001).

Representative Day-28 H&E-stained liver sections are shown in Figure 3C. Histological features were assessed qualitatively, without an injury score or statistical comparison between groups.

### 3.3. Post-Exposure 3-MA Administration Is Associated with Lower Fibrosis-Related Indices

**Figure 4:**
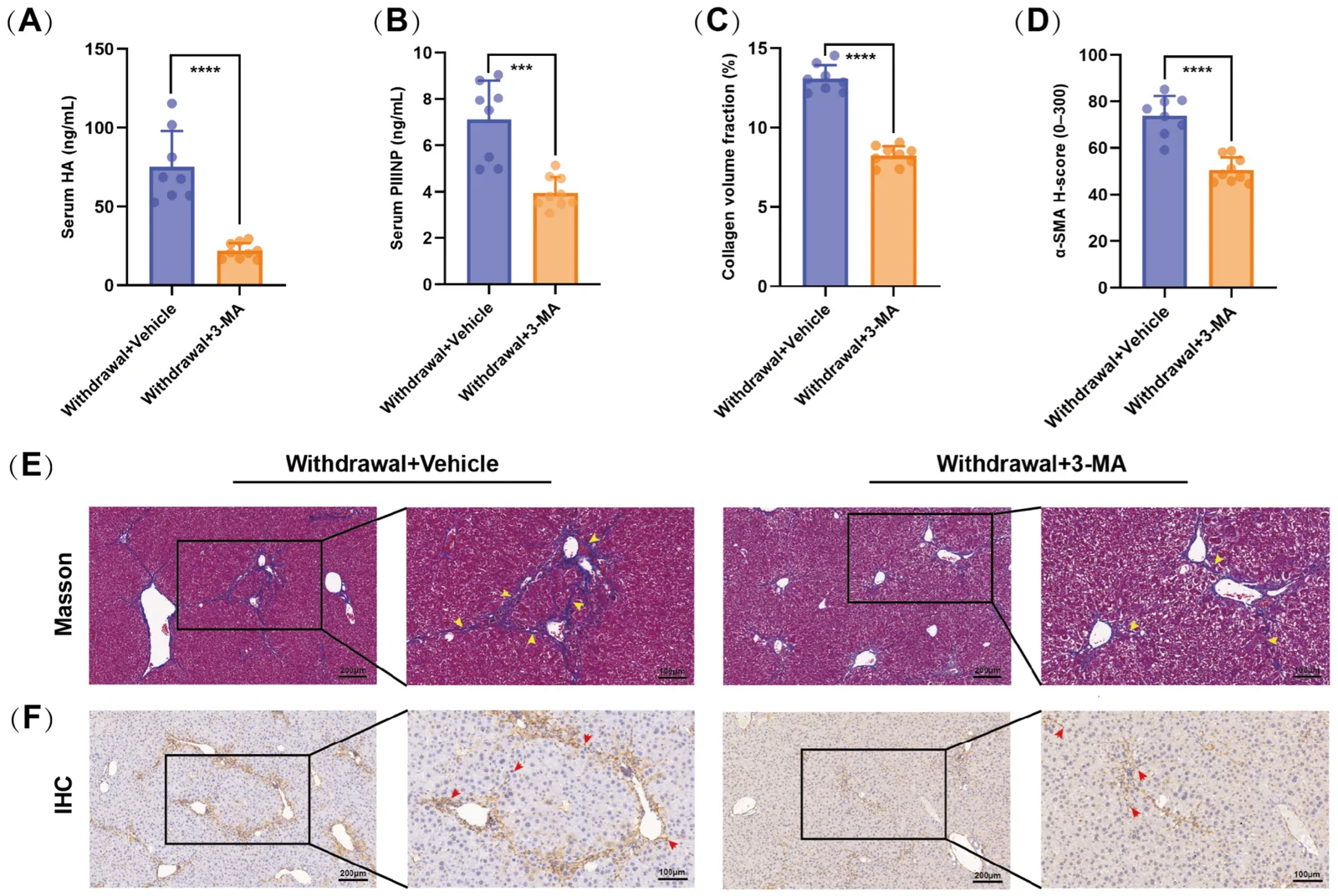
Fibrosis-related serum markers and hepatic histology at Day 28 after arsenic withdrawal. Following sodium arsenite withdrawal, mice received vehicle (Withdrawal + Vehicle, *n* = 8) or 3-MA (Withdrawal + 3-MA, *n* = 9) twice weekly for 4 weeks. (**A**,**B**) Serum hyaluronic acid (HA) and N-terminal propeptide of type III procollagen (PIIINP), respectively. (**C**) Collagen volume fraction quantified from Masson’s trichrome staining. (**D**) Hepatic α-smooth muscle actin (α-SMA) H-score. (**E**,**F**) Representative Masson’s trichrome staining (**E**) and α-SMA immunohistochemistry (**F**); boxed regions are enlarged. Yellow and red arrowheads indicate collagen deposition or fibrous cords and α-SMA-positive staining, respectively. Scale bars: 200 μm for overview images and 100 μm for enlarged images. Data are mean ± SD; each dot represents one mouse. Between-group comparisons used an unpaired two-sided Student’s *t*-test or Mann–Whitney *U* test. *** *p* < 0.001; **** *p* < 0.0001.

At Day 28, serum HA and PIIINP concentrations were lower in the Withdrawal + 3-MA group than in the Withdrawal + Vehicle group (*p* < 0.0001 and *p* < 0.001, respectively; Figure 4A,B). Collagen volume fraction was also lower in the Withdrawal + 3-MA group (*p* < 0.0001; Figure 4C). Representative Masson’s trichrome-stained sections from Withdrawal + Vehicle mice showed prominent collagen deposition and fibrous cords, whereas these features appeared less extensive in sections from Withdrawal + 3-MA mice (Figure 4E).

Representative α-SMA immunohistochemistry likewise showed less prominent immunoreactivity in the Withdrawal + 3-MA group (Figure 4F), accompanied by a lower α-SMA H-score (*p* < 0.0001; Figure 4D). 

### 3.4. Post-Exposure 3-MA Administration Is Associated with Lower Serum TNF-α and Altered Hepatic NF-κB-Related Protein Levels

**Figure 5:**
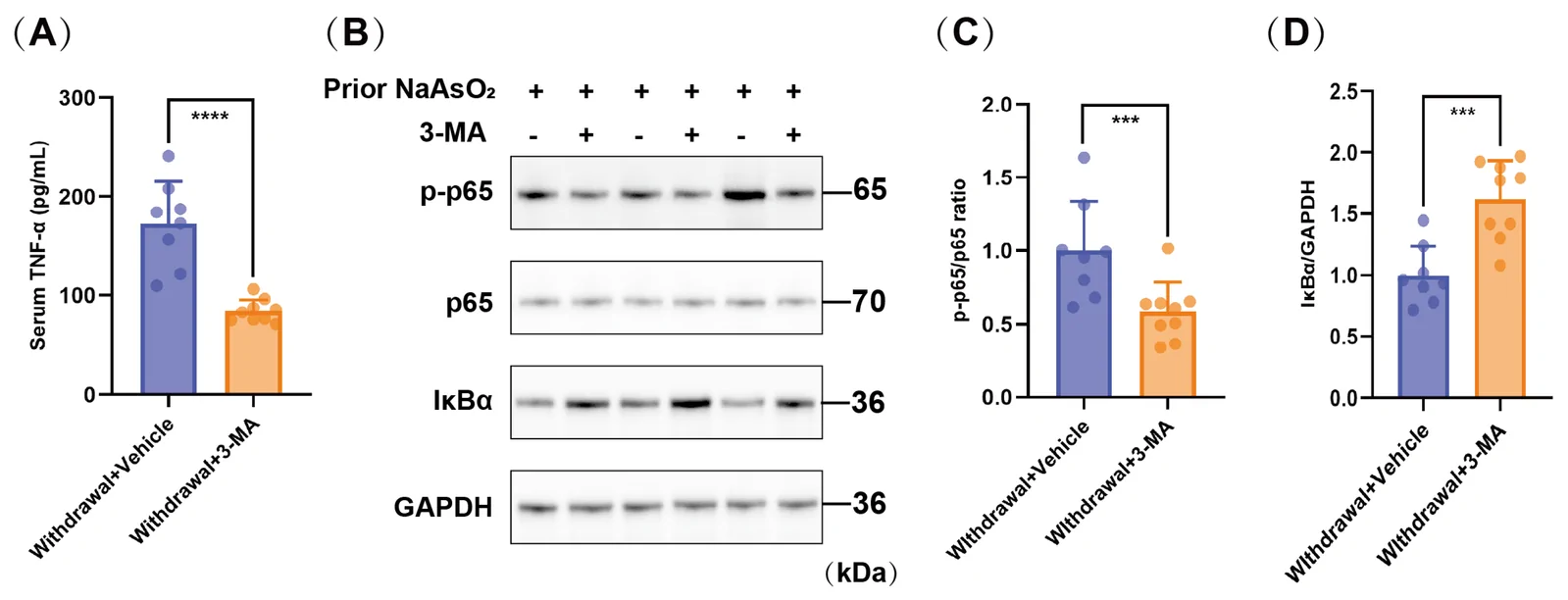
Serum TNF-α and hepatic NF-κB-related proteins at Day 28 after arsenic withdrawal. (**A**) Serum tumor necrosis factor-α (TNF-α). (**B**) Representative immunoblots of phosphorylated p65 (p-p65), total p65, IκBα, and GAPDH. (**C**,**D**) p-p65/p65 and IκBα/GAPDH ratios, respectively. Quantification included all mice, with three representative lanes per group shown in (**B**). Data are mean ± SD; each dot represents one mouse (Withdrawal + Vehicle, *n* = 8; Withdrawal + 3-MA, *n* = 9). Between-group comparisons used an unpaired two-sided Student’s *t*-test or Mann–Whitney *U* test. *** *p* < 0.001; **** *p* < 0.0001.

At Day 28, serum TNF-α was lower in the Withdrawal + 3-MA group than in the Withdrawal + Vehicle group (*p* < 0.0001; Figure 5A). Representative immunoblots of total liver protein extracts showed lower p-p65 band intensity and higher IκBα band intensity in the Withdrawal + 3-MA group (Figure 5B). Quantification across all mice showed a lower p-p65/p65 ratio and higher GAPDH-normalized IκBα abundance in the Withdrawal + 3-MA group (both *p* < 0.001; Figure 5C,D).

### 3.5. Post-Exposure 3-MA Administration Is Associated with Altered Hepatic Autophagy-Related Protein Levels

**Figure 6:**
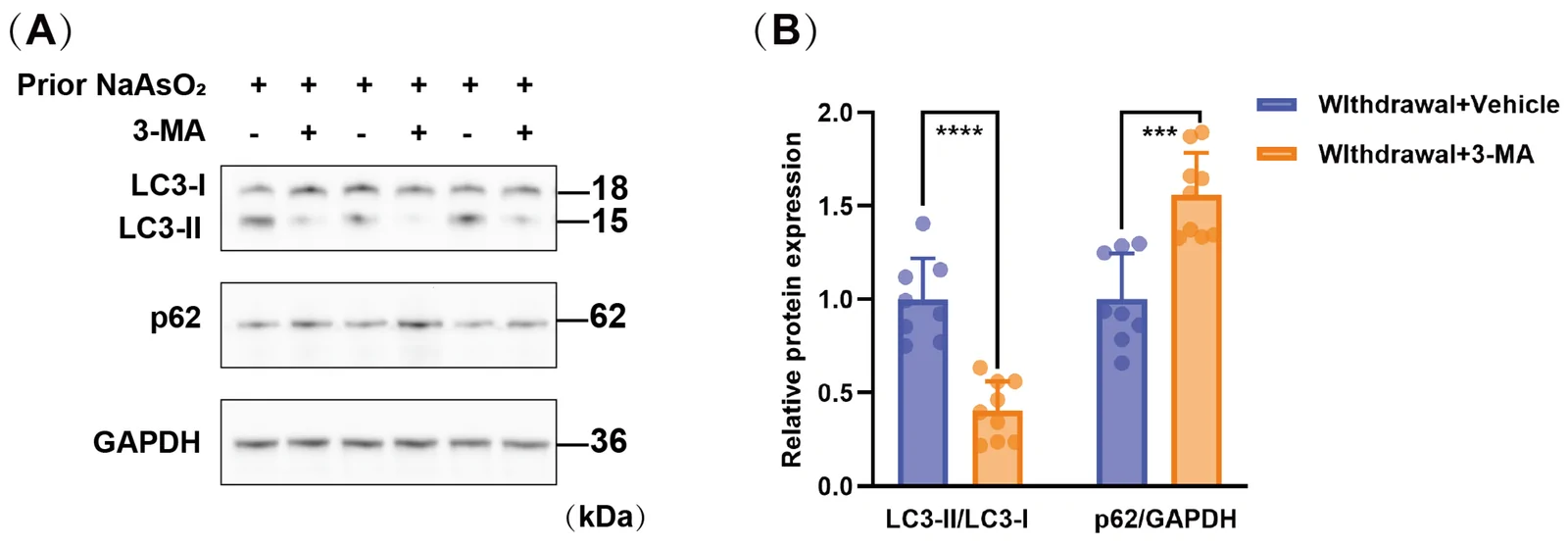
Hepatic autophagy-related proteins at Day 28 after arsenic withdrawal. (**A**) Representative immunoblots of LC3-I, LC3-II, p62, and GAPDH in total liver protein extracts. (**B**) LC3-II/LC3-I and p62/GAPDH ratios. Quantification included all mice, with three representative lanes per group shown in (**A**). Data are mean ± SD; each dot represents one mouse (Withdrawal + Vehicle, *n* = 8; Withdrawal + 3-MA, *n* = 9). Between-group comparisons used an unpaired two-sided Student’s *t*-test or Mann–Whitney *U* test, as appropriate. *** *p* < 0.001; **** *p* < 0.0001.

At Day 28, representative immunoblots showed lower LC3-II and higher p62 band intensities in the Withdrawal + 3-MA group (Figure 6A). Quantification across all mice showed a lower LC3-II/LC3-I ratio (*p* < 0.0001) and a higher p62/GAPDH ratio (*p* < 0.001; Figure 6B). 

## 4. Discussion

In previously exposed female mice, post-exposure 3-MA administration was associated with lower Day-28 serum aminotransferase and fibrosis-related indices than vehicle administration. These differences were accompanied by lower TNF-α, a lower p-p65/p65 ratio, higher IκBα, and altered LC3-II/LC3-I and p62. The study describes post-exposure phenotypic differences and associated protein changes, without establishing the pathway responsible.

Removal of a hepatic insult can permit fibrosis regression, although recovery varies across injury contexts [2,5,6]. Here, 3-MA administration began after 20 weeks of sodium arsenite exposure, and both intervention groups subsequently received arsenic-free water. The vehicle group provided a comparator for the post-exposure course under vehicle administration. However, the absence of concurrent unexposed mice prevented assessment of whether endpoint values had returned to the unexposed range. In addition, no unexposed group received 3-MA, preventing assessment of its independent hepatotoxic or hepatoprotective effects in the absence of arsenic exposure. Because all intervention animals stopped receiving arsenic, the comparison also does not isolate the effect of exposure withdrawal itself. Moreover, Week-20 and Day-28 liver samples came from different animals, so the endpoint findings do not establish within-animal fibrosis regression.

Baseline ALT was numerically lower in the Withdrawal + 3-MA group, which complicates interpretation of the unadjusted endpoint comparison. The Day-28 ALT difference persisted after adjustment for Day-0 ALT. For AST, the baseline-by-group interaction argued against reporting a single common-slope ANCOVA estimate. The longitudinal and change-score analyses showed a consistent direction, but change-score analysis is not equivalent to covariate adjustment. These analyses were post hoc, and the small sample leaves uncertainty about the magnitude and stability of the group differences.

Inflammation links hepatocellular injury with fibrogenesis. Hepatocytes, Kupffer cells, recruited immune cells, and activated stellate cells can produce mediators that sustain injury and extracellular-matrix deposition [5,30,31,32]. NF-κB regulates cytokine production, cell survival, immune-cell recruitment, and tissue remodeling [16,17]. Lower TNF-α and p-p65/p65, together with higher IκBα, were consistent with differences in inflammatory signaling between the post-exposure groups. These whole-liver measurements do not identify the responding cell populations or establish whether the protein changes preceded the biochemical differences.

Autophagy has cell-type- and stage-dependent functions in liver disease. Basal autophagy can protect hepatocytes, whereas autophagy may support hepatic stellate-cell activation in some fibrotic settings [21,33,34,35]. In mouse models, loss of autophagy in CD4 T cells promoted liver fibrosis through type 3 inflammation [36]. Arsenic-related alterations in autophagy have been reported in hepatic stellate cells [14], while disruption of the autophagy–lysosome pathway has been demonstrated in cultured dendritic cells [37]. In an arsenic trioxide model, autophagy–inflammasome signaling was implicated in liver inflammation [26]. 

Arsenic can also alter autophagy, lysosomal function, and inflammasome-related pathways [14,26,37]. Withdrawal + 3-MA mice had a lower LC3-II/LC3-I ratio and higher p62 abundance, but these static measurements do not establish autophagic flux [27]. Furthermore, 3-MA affects class I and class III phosphoinositide 3-kinases in a context-dependent manner [28]. PI3K activity, PI3K phosphorylation, and PI3K lipid products were not measured in this study. Consequently, the effects of 3-MA on PI3K signaling under the present experimental conditions were not established, and the observed hepatic differences cannot be attributed specifically to PI3K or autophagy inhibition.

Several additional limitations remain. Blood and hepatic arsenic concentrations were not measured, precluding assessment of internal arsenic burden and its changes after exposure withdrawal. Consequently, we could not distinguish the effects of retained arsenic from injury persisting after clearance, or determine whether the differences in hepatic outcomes between the intervention groups were accompanied by differences in arsenic burden. Water consumption was measured at the cage level, and arsenic intake was estimated using cage-derived mean water intake and group mean body weight. Individual water consumption and arsenic intake were not determined; therefore, variation in exposure between mice could not be quantified. Week-20 characterization included only three mice per group. Sex-related differences in tissue arsenic distribution have been reported in mice, with patterns varying by genotype and exposure level [38]. Estrogen receptor 1 signaling in hepatic stellate cells has also been shown to limit fibrosis in a diet-induced mouse model [39]. Only female mice were studied here, precluding direct assessment of sex differences in arsenic-associated liver injury or the response to 3-MA and limiting generalization to males. Estrous cycles were not monitored, so potential cycle-related influences could not be evaluated. H&E findings were qualitative and do not establish a quantified reduction in histological injury. Inflammatory assessment included a limited set of markers, and no pathway-rescue or genetic experiments were performed. Independent studies with baseline-stratified randomization, concurrent unexposed controls, and functional pathway measurements are needed to test the reproducibility and biological basis of these findings.

## 5. Conclusions

During the 4 weeks after sodium arsenite withdrawal, 3-MA administration was associated with more favorable serum ALT and AST trajectories in female mice. Compared to vehicle, 3-MA was also associated with lower Day-28 serum fibrosis-related markers, collagen volume fraction, and α-SMA immunoreactivity. These differences were accompanied by lower serum TNF-α and changes in hepatic NF-κB- and autophagy-related proteins. The findings support further investigation of post-exposure interventions for arsenic-associated liver injury. However, they do not establish an autophagy-dependent mechanism or demonstrate within-animal fibrosis regression. Further studies are needed to clarify the underlying mechanisms, assess reproducibility, and determine whether these differences persist over longer follow-up.

## Data Availability

The original contributions presented in this study are included in the article/Supplementary Material. Further inquiries can be directed to the corresponding author.

## Supplementary Materials

The following supporting information can be downloaded at: https://www.mdpi.com/article/10.3390/toxics14100873/s1, Supplementary Data S1: Numerical source data; Supplementary File S1: Western blot source images for Figure 5 and Figure 6.
