# Supplementary material for: Post-Exposure 3-Methyladenine Is Associated with Lower Indices of Hepatic Injury and Fibrotic Remodeling After Chronic Sodium Arsenite Exposure in Mice
Source: Toxics. 2026 Sep 30;14(10):873. doi: 10.3390/toxics14100873 (PMC13709370; doi:10.3390/toxics14100873)
Supplement: Supplementary file 1 [file toxics-14-00873-s001.zip › toxics-4580157-supplementary.pdf]

Supplementary File S1

Western blot source images for Figures 5 and 6

Liver samples collected at Day 28 after sodium arsenite withdrawal. The source images cover all 17 mice: Withdrawal+Vehicle (*n* = 8) and Withdrawal+3-MA (*n* = 9).

Sample identity and loading order

Lane*	Set 1	Group	Set 2	Group
1	P321	V	P325	V
2	P329	3-MA	P333	3-MA
3	P322	V	P326	V
4	P330	3-MA	P334	3-MA
5	P323	V	P327	V
6	P331	3-MA	P335	3-MA
7	P324	V	P328	V
8	P332	3-MA	P336	3-MA
9	—	—	P337	3-MA

*Sample lanes are numbered from left to right, excluding marker lanes (M).

V = Withdrawal+Vehicle; 3-MA = Withdrawal+3-MA.

Relationship to the manuscript

Figure 5B: set 1, lanes 1–6 (P321, P329, P322, P330, P323, P331) for p-p65, p65, IκBα and GAPDH.

Figure 6A: set 2, lanes 1–6 (P325, P333, P326, P334, P327, P335) for LC3, p62 and GAPDH.

X marks a lane not displayed in the representative manuscript panel. It does not indicate exclusion from quantification. All sample lanes remain visible in this file.

p-p65 sample set 1

Figure 5C: p-p65/p65

V = Withdrawal+Vehicle; 3-MA = Withdrawal+3-MA; M = marker.

Chemiluminescence

p-p65 65 kDa

p-p65/set1_chemiluminescence.tif

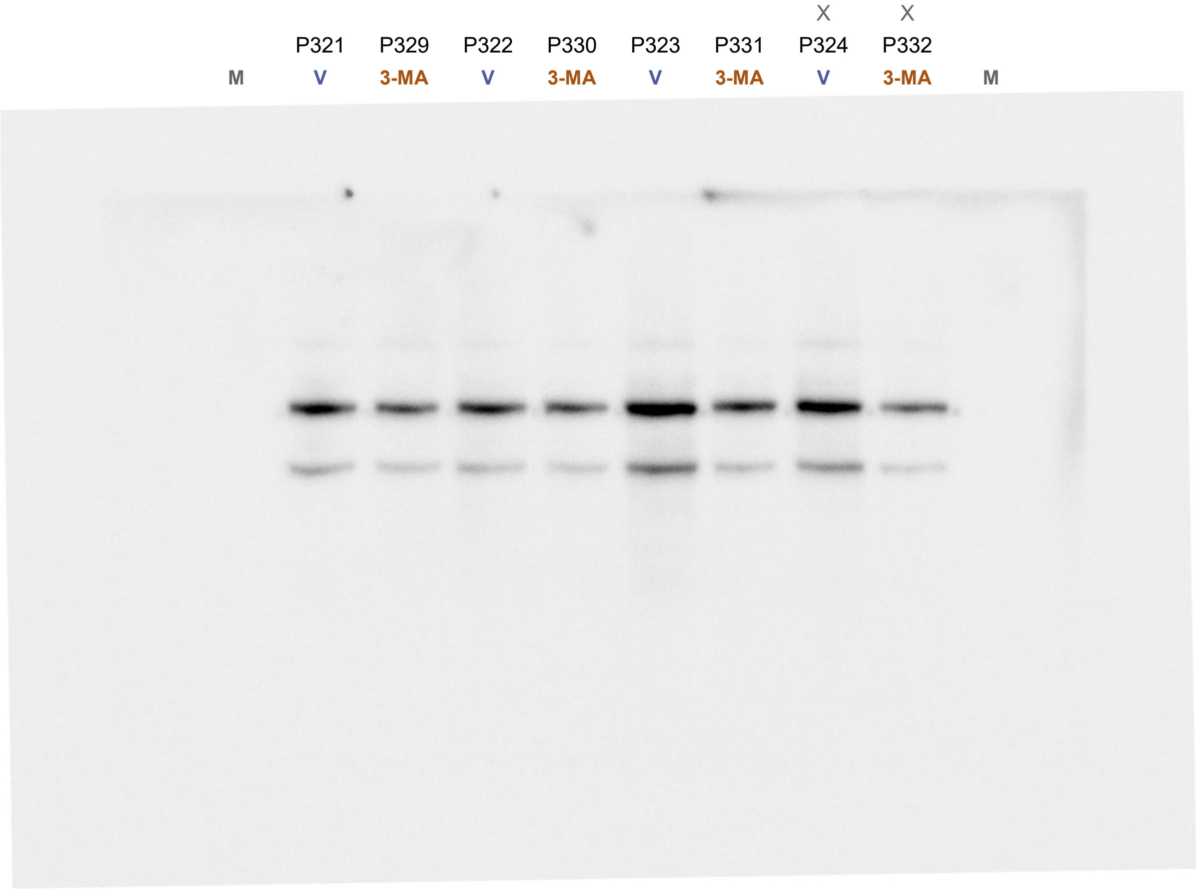

Representative panel: Figure 5B, sample lanes 1–6.

X = not displayed in the representative panel; all lanes included in the reported quantification.

White light

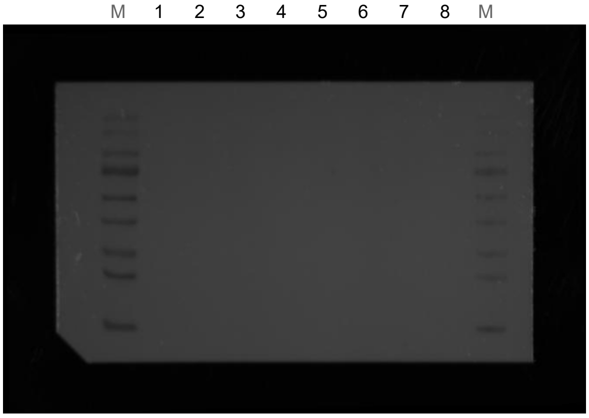

Merge (supplied overlay)

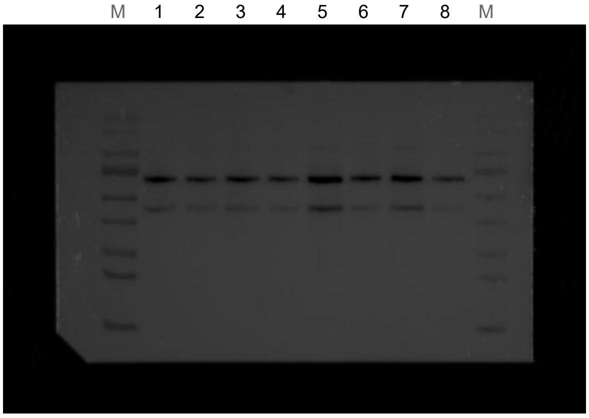

Lower-panel lane numbers refer to the mouse IDs above. No image fields have been cropped here.

p-p65 sample set 2

Figure 5C: p-p65/p65

V = Withdrawal+Vehicle; 3-MA = Withdrawal+3-MA; M = marker.

Chemiluminescence

p-p65 65 kDa

p-p65/set2_chemiluminescence.tif

M	X P325 V	X P333 3-MA	X P326 V	X P334 3-MA	X P327 V	X P335 3-MA	X P328 V	X P336 3-MA	X P337 3-MA	M

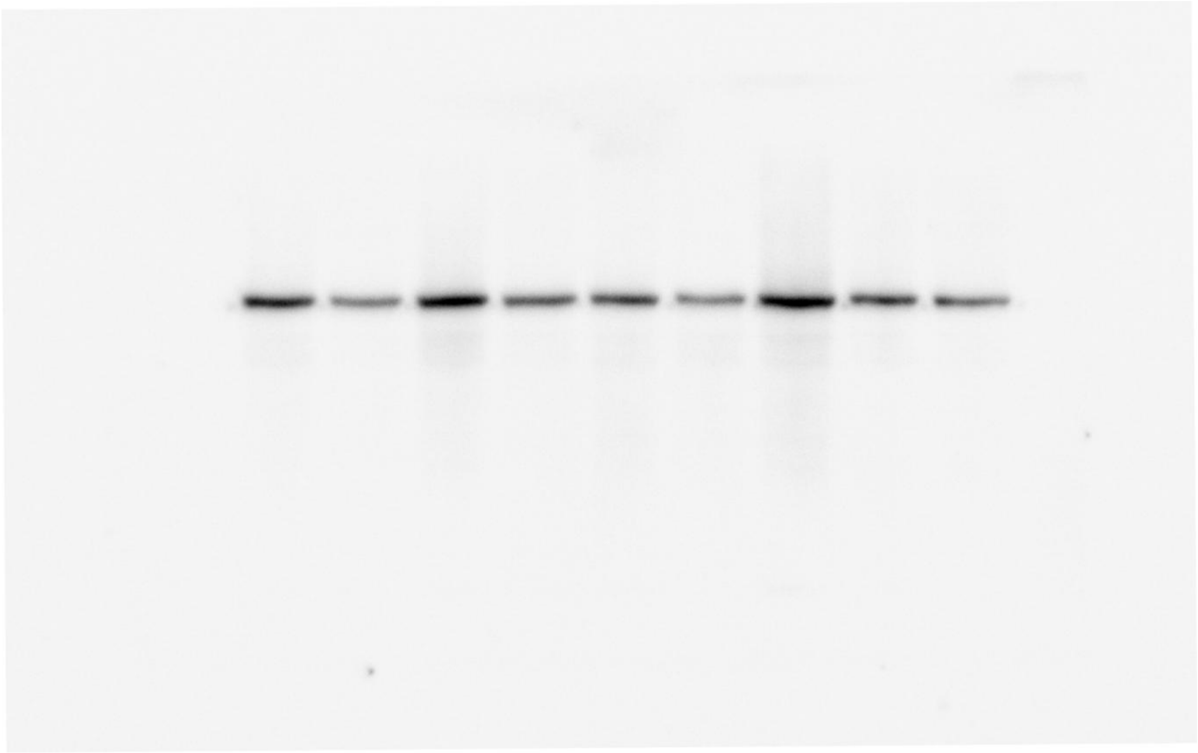

This set supports quantification; not displayed in Figure 5B.

X = not displayed in the representative panel; all lanes included in the reported quantification.

White light

M 1 2 3 4 5 6 7 8 9 M

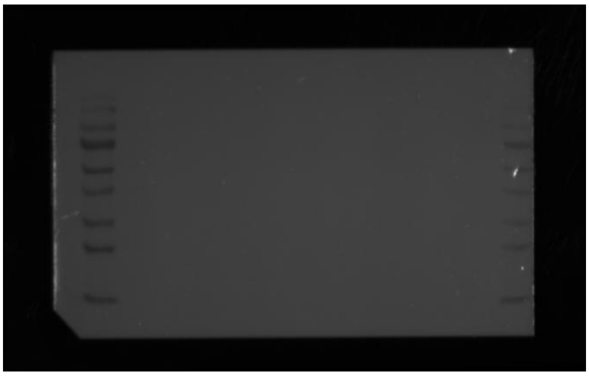

Merge (supplied overlay)

M 1 2 3 4 5 6 7 8 9 M

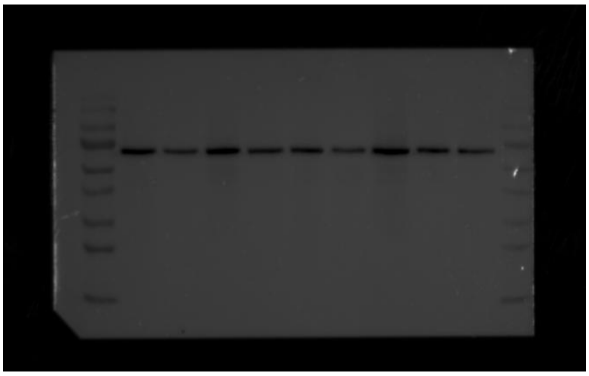

Lower-panel lane numbers refer to the mouse IDs above. No image fields have been cropped here.

p65 sample set 1

Figure 5C: p-p65/p65

V = Withdrawal+Vehicle; 3-MA = Withdrawal+3-MA; M = marker.

Chemiluminescence

p65 70 kDa

p65/set1_chemiluminescence.tif

							X	X	
	P321	P329	P322	P330	P323	P331	P324	P332	
**M**	**V**	**3-MA**	**V**	**3-MA**	**V**	**3-MA**	**V**	**3-MA**	**M**

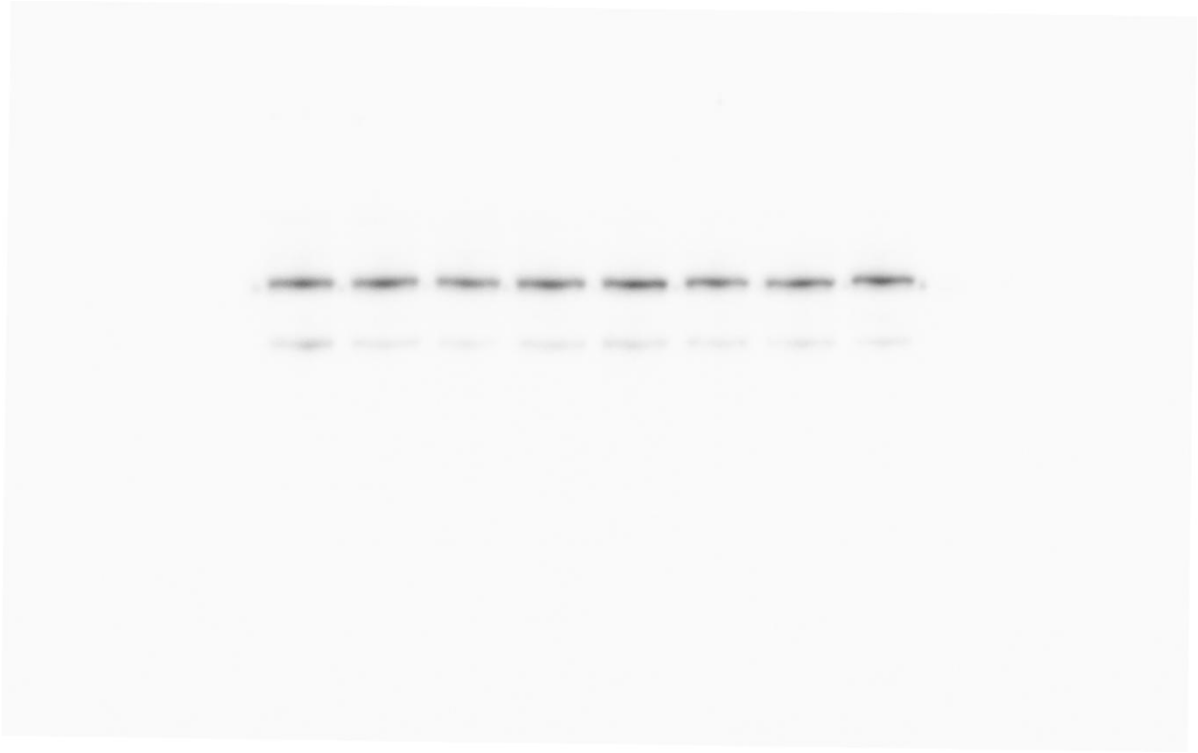

Representative panel: Figure 5B, sample lanes 1–6.

X = not displayed in the representative panel; all lanes included in the reported quantification.

White light

M	1	2	3	4	5	6	7	8	M

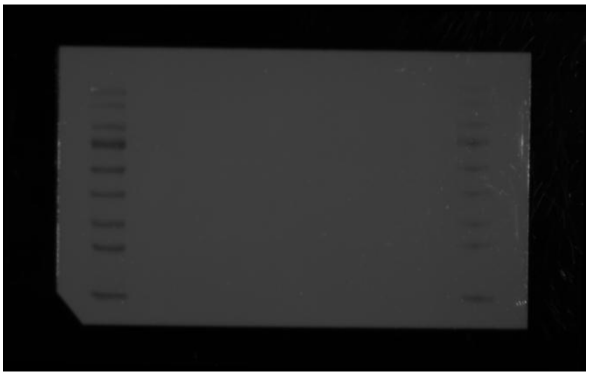

Merge (supplied overlay)

M	1	2	3	4	5	6	7	8	M

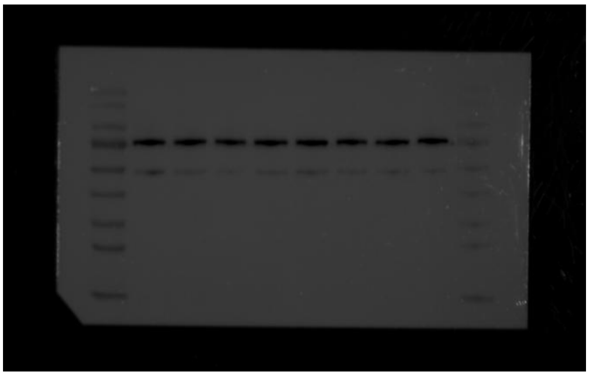

Lower-panel lane numbers refer to the mouse IDs above. No image fields have been cropped here.

p65 sample set 2

Figure 5C: p-p65/p65

V = Withdrawal+Vehicle; 3-MA = Withdrawal+3-MA; M = marker.

Chemiluminescence

p65 70 kDa

p65/set2_chemiluminescence.tif

	X	X	X	X	X	X	X	X	X	
	P325	P333	P326	P334	P327	P335	P328	P336	P337	
**M**	**V**	**3-MA**	**V**	**3-MA**	**V**	**3-MA**	**V**	**3-MA**	**3-MA**	**M**

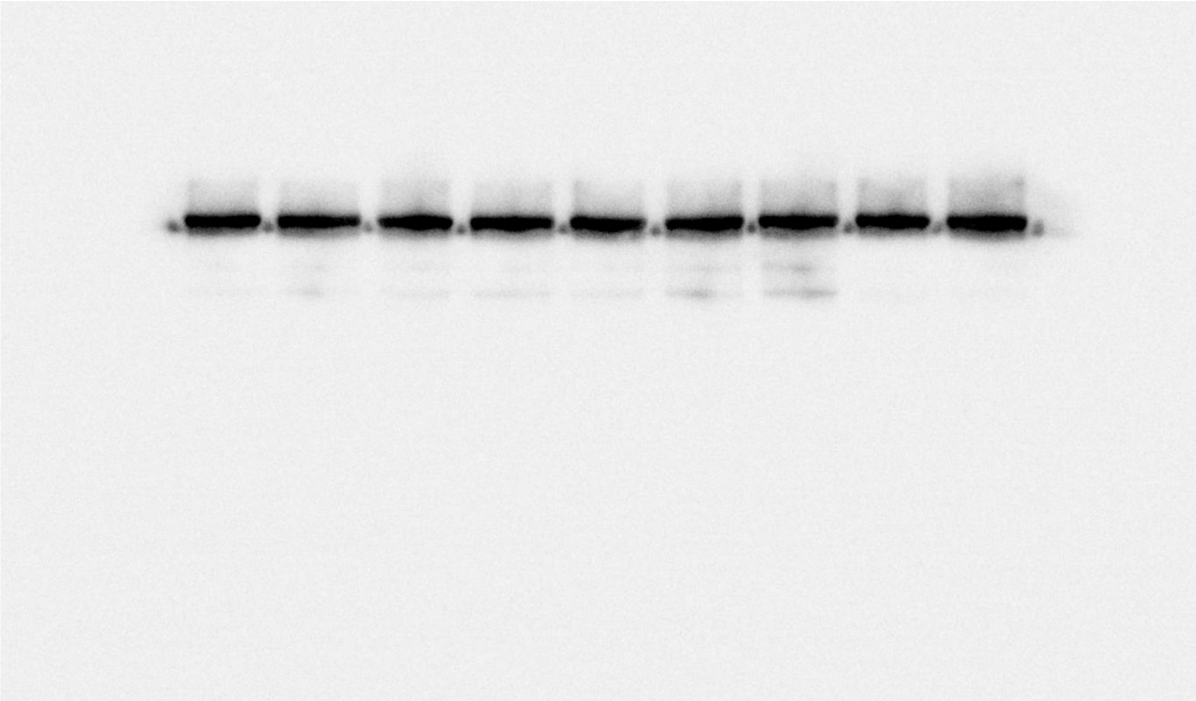

This set supports quantification; not displayed in Figure 5B.

X = not displayed in the representative panel; all lanes included in the reported quantification.

White light

M 1 2 3 4 5 6 7 8 9 M

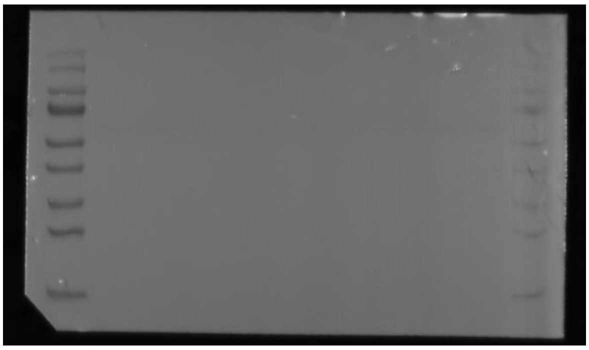

Merge (supplied overlay)

M 1 2 3 4 5 6 7 8 9 M

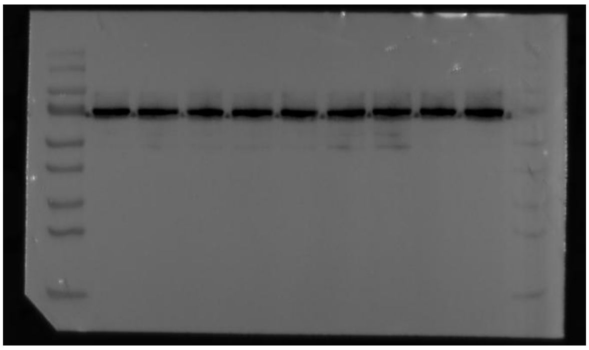

Lower-panel lane numbers refer to the mouse IDs above. No image fields have been cropped here.

IκBα sample set 1

Figure 5D: IκBα/GAPDH

V = Withdrawal+Vehicle; 3-MA = Withdrawal+3-MA; M = marker.

Chemiluminescence

IκBα 36 kDa

IkBa/set1_chemiluminescence.tif

							X	X	
	P321	P329	P322	P330	P323	P331	P324	P332	
**M**	**V**	**3-MA**	**V**	**3-MA**	**V**	**3-MA**	**V**	**3-MA**	**M**

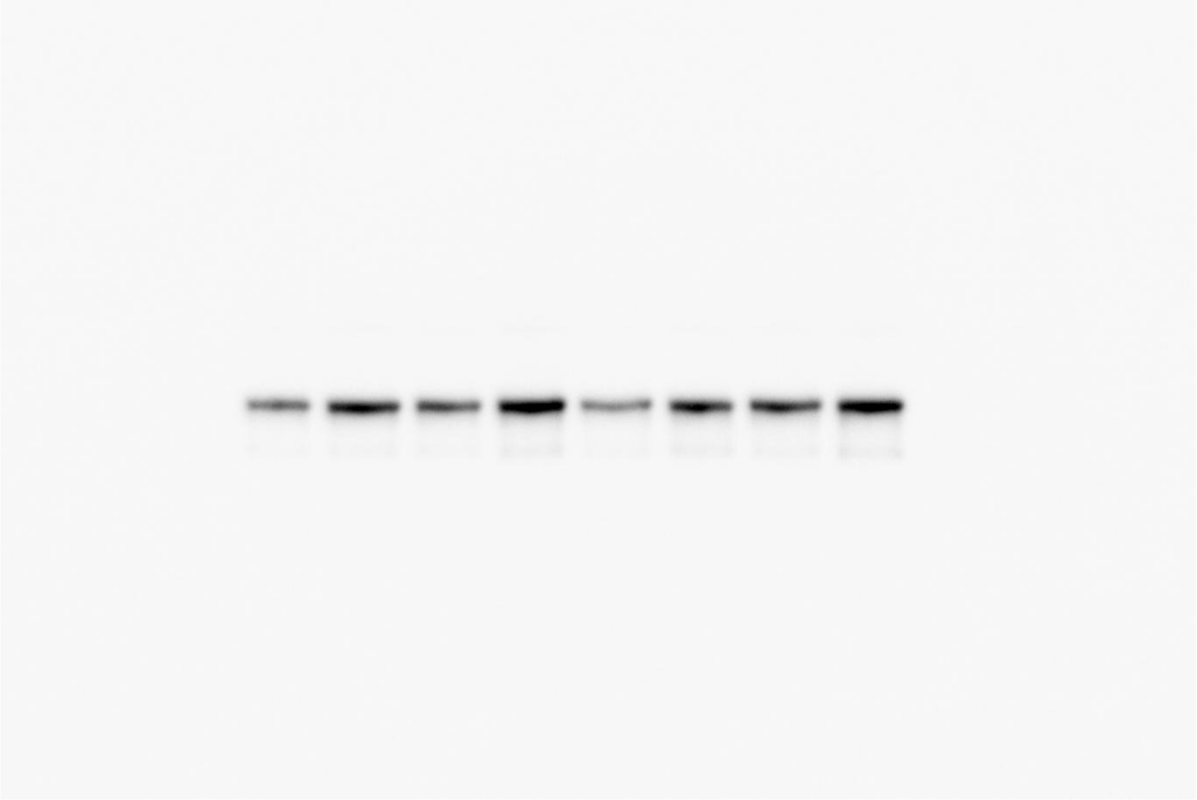

Representative panel: Figure 5B, sample lanes 1–6.

X = not displayed in the representative panel; all lanes included in the reported quantification.

White light

M 1 2 3 4 5 6 7 8 M

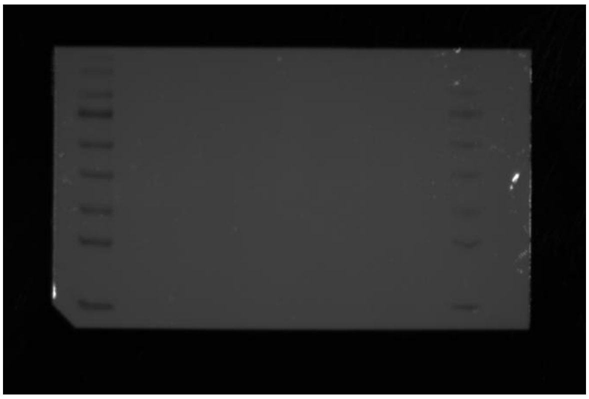

Merge (supplied overlay)

M 1 2 3 4 5 6 7 8 M

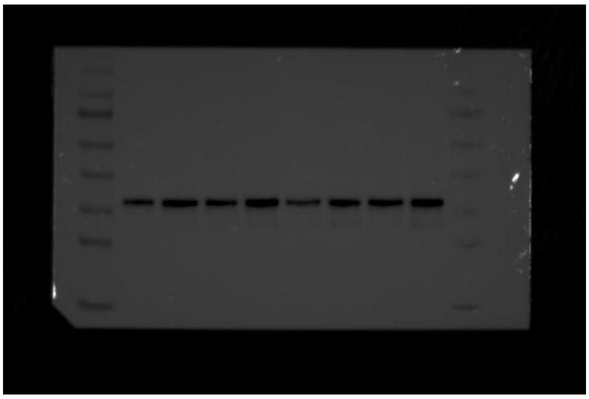

Lower-panel lane numbers refer to the mouse IDs above. No image fields have been cropped here.

IκBα sample set 2

Figure 5D: IκBα/GAPDH

V = Withdrawal+Vehicle; 3-MA = Withdrawal+3-MA; M = marker.

Chemiluminescence

IκBα 36 kDa

IkBa/set2_chemiluminescence.tif

	X	X	X	X	X	X	X	X	X	
	P325	P333	P326	P334	P327	P335	P328	P336	P337	
M	V	3-MA	V	3-MA	V	3-MA	V	3-MA	3-MA	M

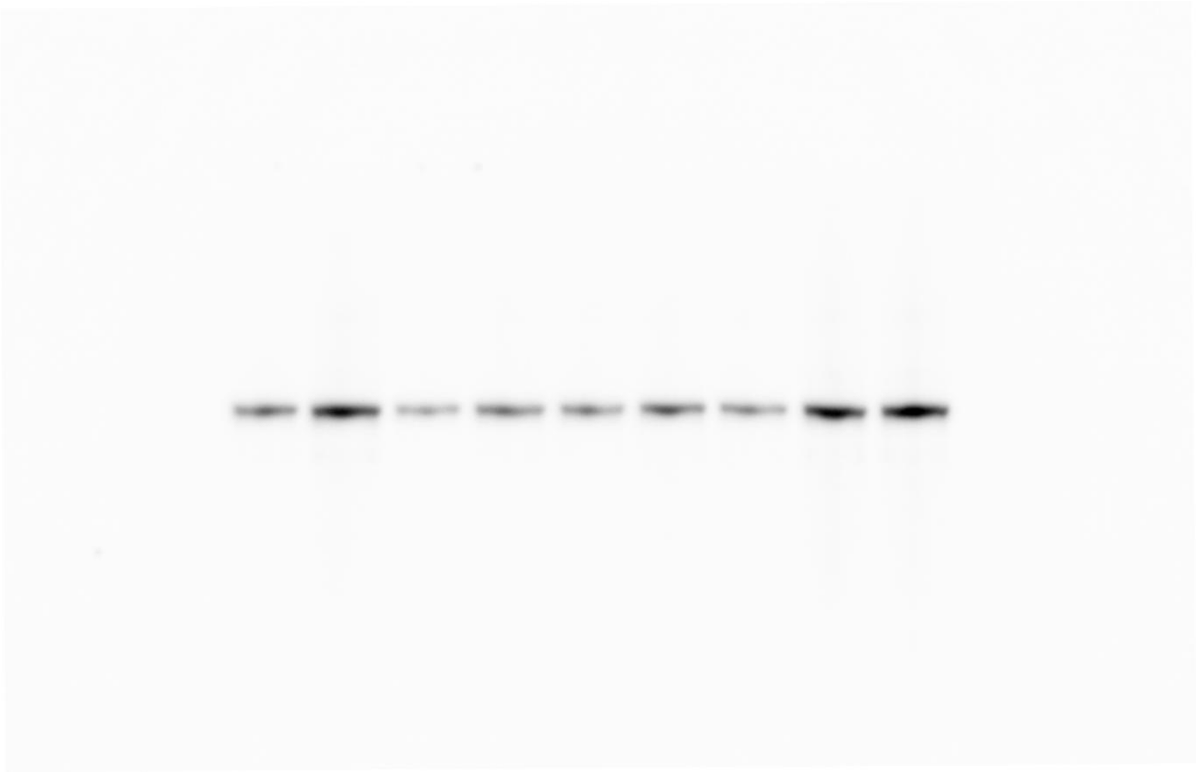

This set supports quantification; not displayed in Figure 5B.

X = not displayed in the representative panel; all lanes included in the reported quantification.

White light

M 1 2 3 4 5 6 7 8 9 M

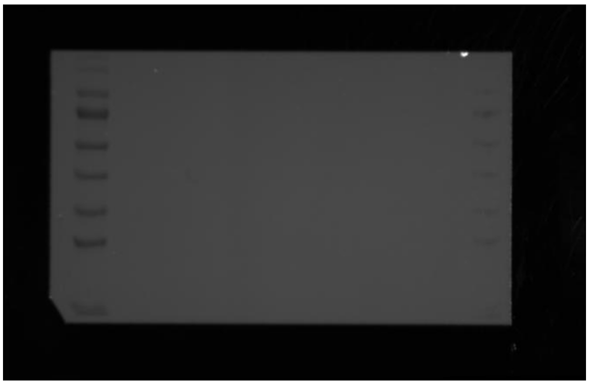

Merge (supplied overlay)

M 1 2 3 4 5 6 7 8 9 M

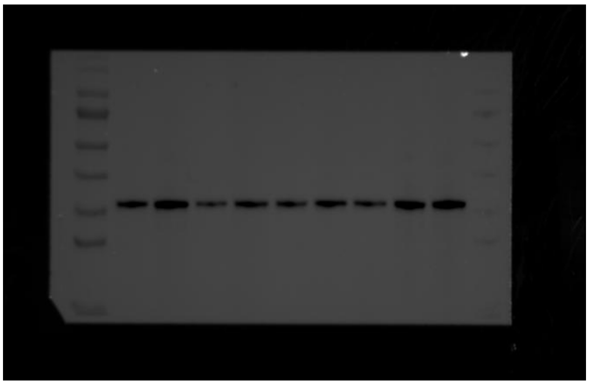

Lower-panel lane numbers refer to the mouse IDs above. No image fields have been cropped here.

LC3-I / LC3-II sample set 1

Figure 6B: LC3-II/LC3-I

V = Withdrawal+Vehicle; 3-MA = Withdrawal+3-MA; M = marker.

Chemiluminescence

LC3-I 18 kDa; LC3-II 15 kDa

LC3/set1_chemiluminescence.tif

	X	X	X	X	X	X	X	X	
	P321	P329	P322	P330	P323	P331	P324	P332	
**M**	**V**	**3-MA**	**V**	**3-MA**	**V**	**3-MA**	**V**	**3-MA**	**M**

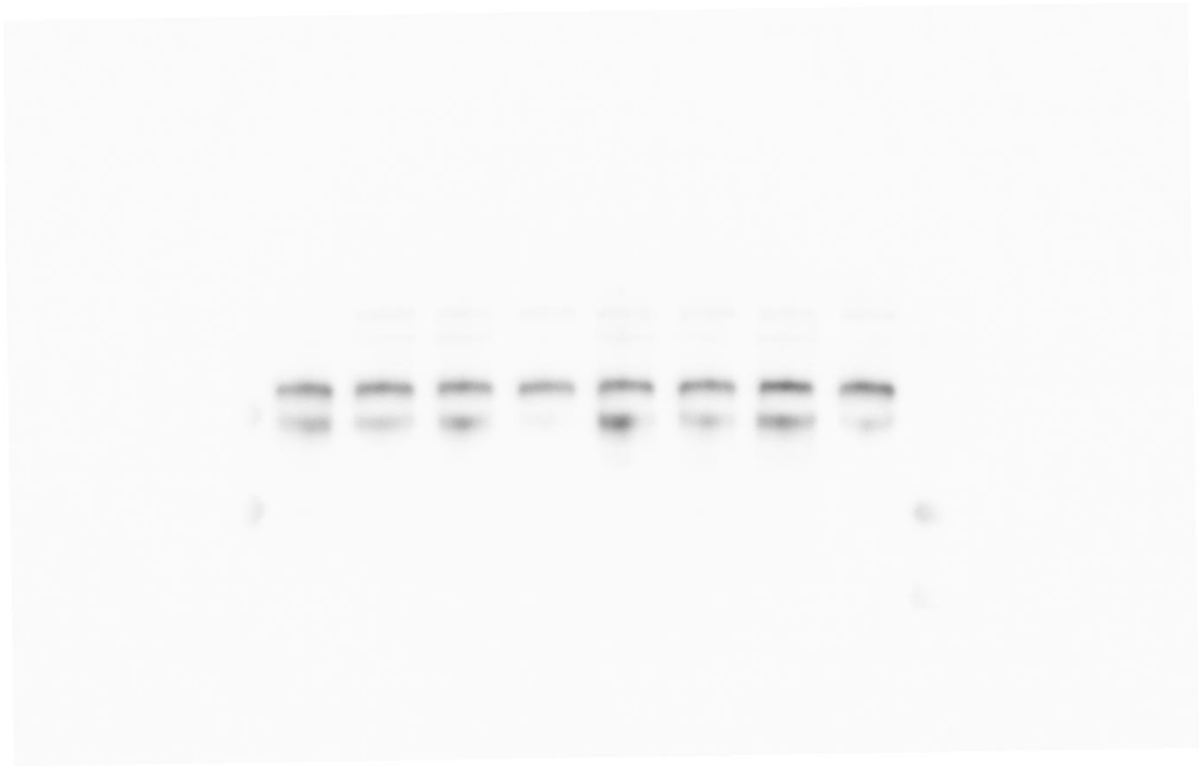

This set supports quantification; not displayed in Figure 6A.

X = not displayed in the representative panel; all lanes included in the reported quantification.

White light

M 1 2 3 4 5 6 7 8 M

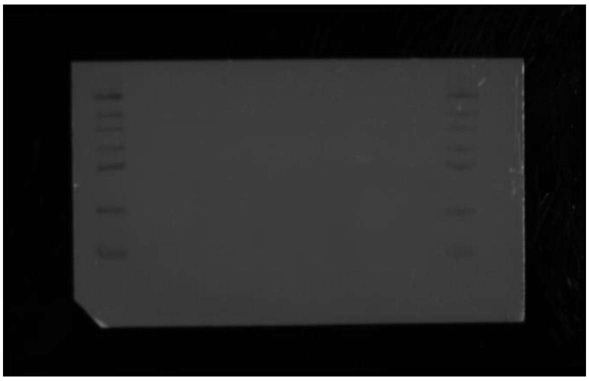

Merge (supplied overlay)

M 1 2 3 4 5 6 7 8 M

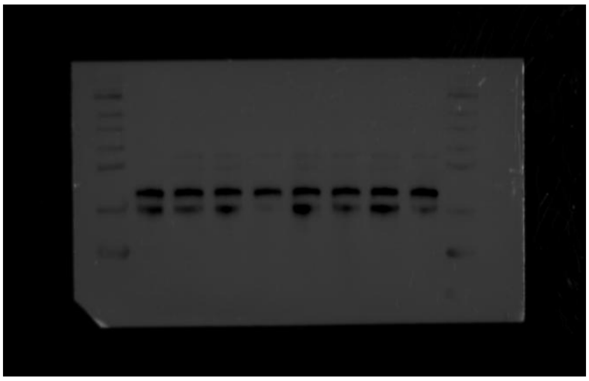

Lower-panel lane numbers refer to the mouse IDs above. No image fields have been cropped here.

LC3-I / LC3-II sample set 2

Figure 6B: LC3-II/LC3-I

V = Withdrawal+Vehicle; 3-MA = Withdrawal+3-MA; M = marker.

Chemiluminescence

LC3-I 18 kDa; LC3-II 15 kDa

LC3/set2_chemiluminescence.tif

							X	X	X	
	P325	P333	P326	P334	P327	P335	P328	P336	P337	
M	V	3-MA	V	3-MA	V	3-MA	V	3-MA	3-MA	M

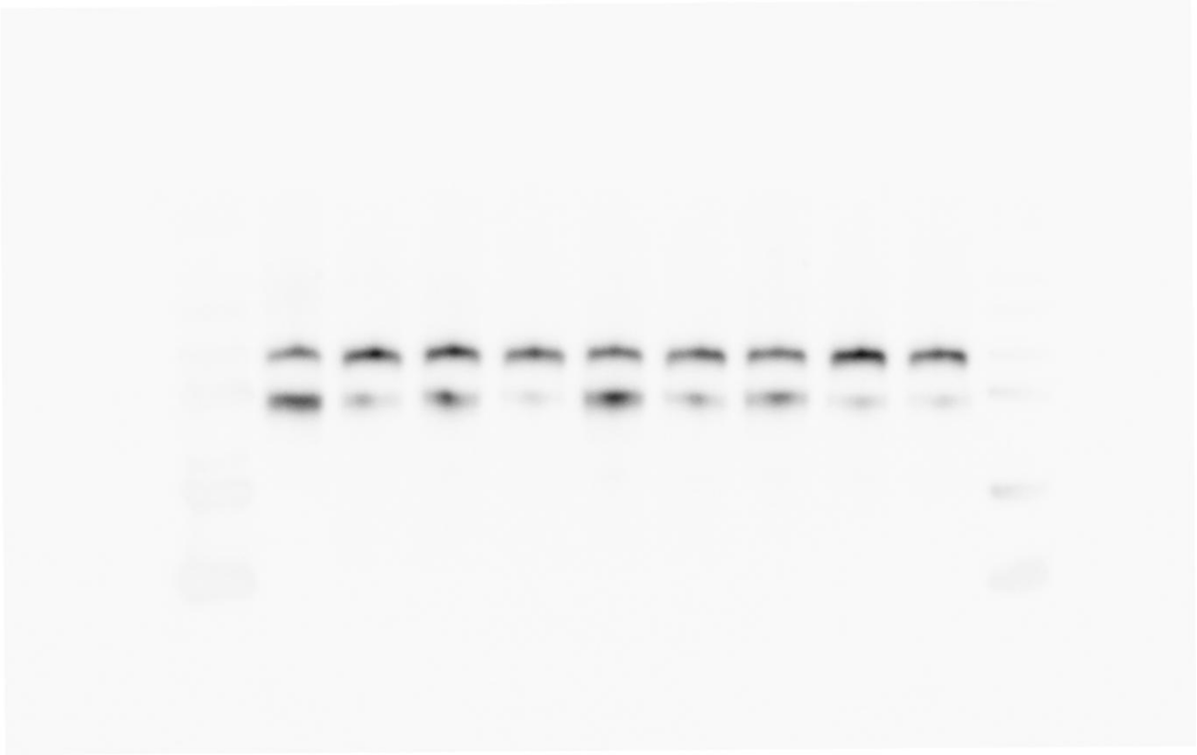

Representative panel: Figure 6A, sample lanes 1–6.

X = not displayed in the representative panel; all lanes included in the reported quantification.

White light

M 1 2 3 4 5 6 7 8 9 M

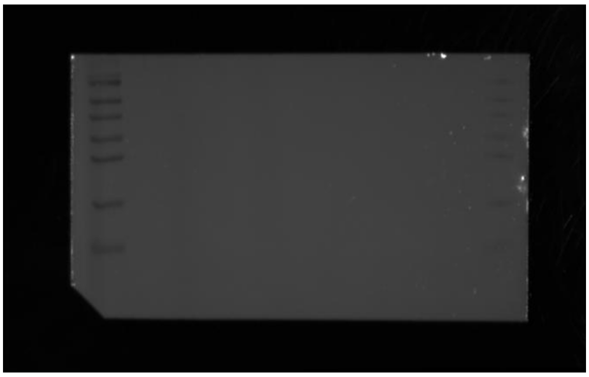

Merge (supplied overlay)

M 1 2 3 4 5 6 7 8 9 M

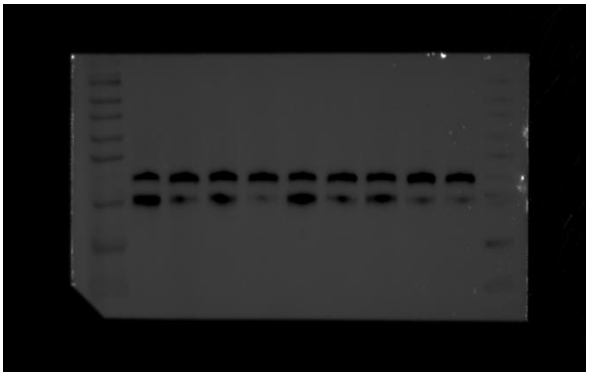

Lower-panel lane numbers refer to the mouse IDs above. No image fields have been cropped here.

p62 sample set 1

Figure 6B: p62/GAPDH

V = Withdrawal+Vehicle; 3-MA = Withdrawal+3-MA; M = marker.

Chemiluminescence

p62 62 kDa

p62/set1_chemiluminescence.tif

	X	X	X	X	X	X	X	X	
	P321	P329	P322	P330	P323	P331	P324	P332	
**M**	**V**	**3-MA**	**V**	**3-MA**	**V**	**3-MA**	**V**	**3-MA**	**M**

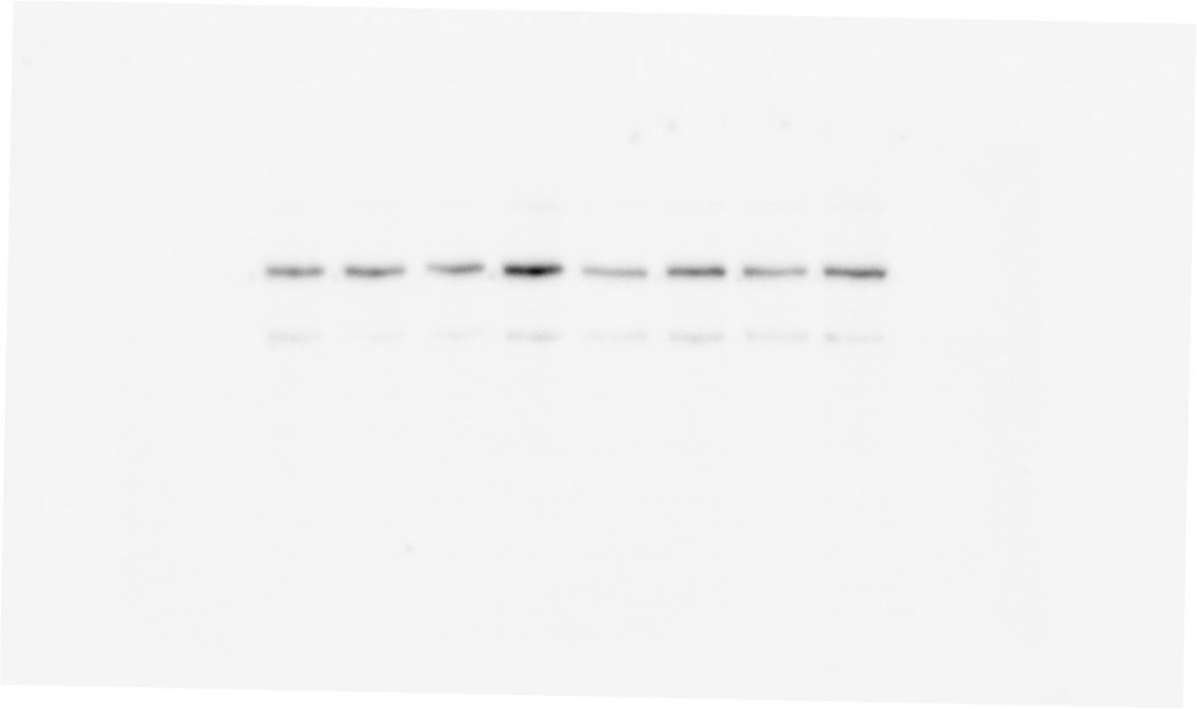

This set supports quantification; not displayed in Figure 6A.

X = not displayed in the representative panel; all lanes included in the reported quantification.

White light

M 1 2 3 4 5 6 7 8 M

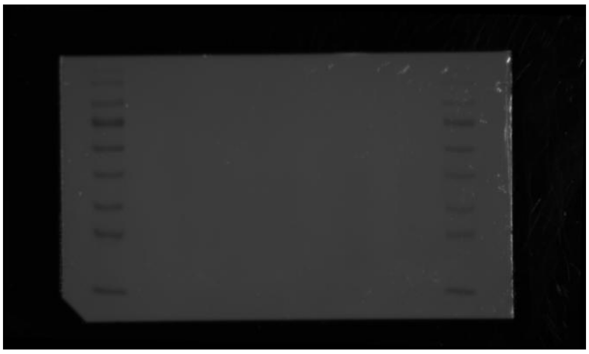

Merge (supplied overlay)

M 1 2 3 4 5 6 7 8 M

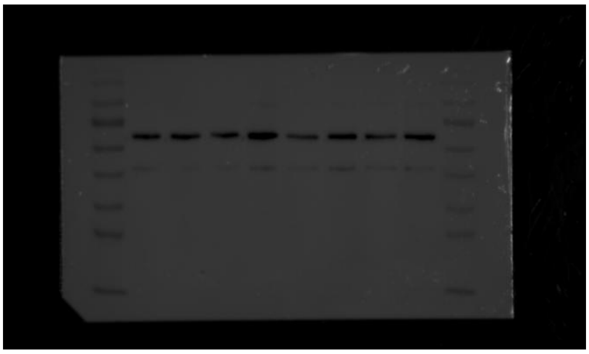

Lower-panel lane numbers refer to the mouse IDs above. No image fields have been cropped here.

p62 sample set 2

Figure 6B: p62/GAPDH

V = Withdrawal+Vehicle; 3-MA = Withdrawal+3-MA; M = marker.

Chemiluminescence

p62 62 kDa

p62/set2_chemiluminescence.tif

							X	X	X	
	P325	P333	P326	P334	P327	P335	P328	P336	P337	
M	V	3-MA	V	3-MA	V	3-MA	V	3-MA	3-MA	M

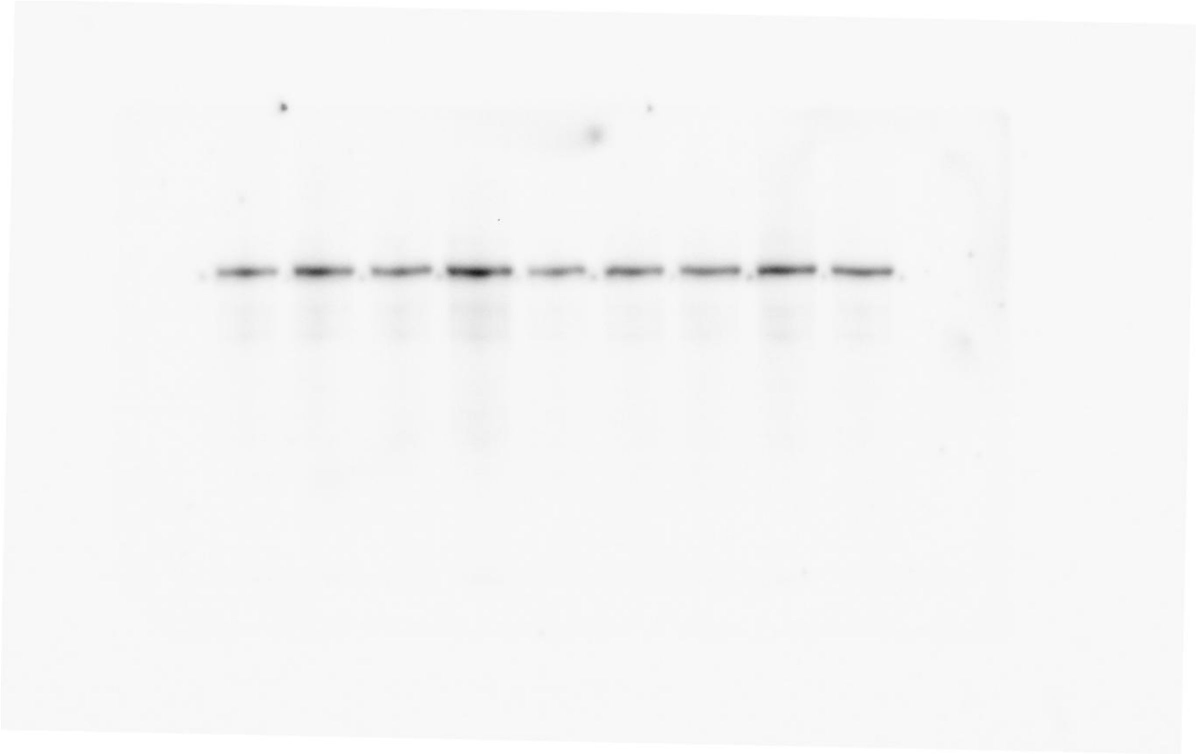

Representative panel: Figure 6A, sample lanes 1–6.

X = not displayed in the representative panel; all lanes included in the reported quantification.

White light

M 1 2 3 4 5 6 7 8 9 M

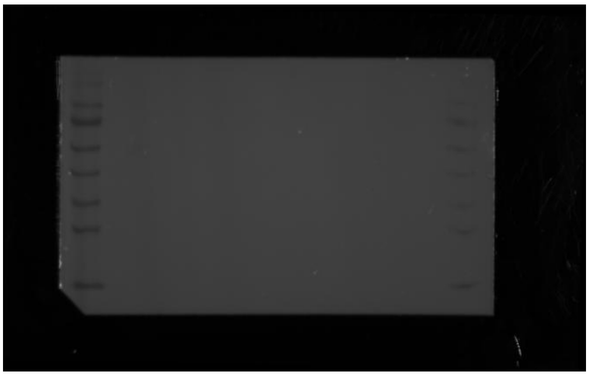

Merge (supplied overlay)

M 1 2 3 4 5 6 7 8 9 M

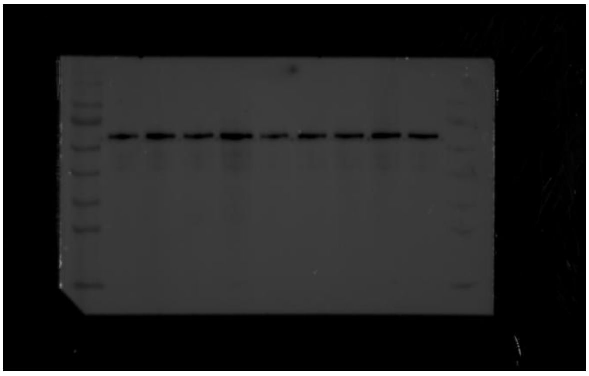

Lower-panel lane numbers refer to the mouse IDs above. No image fields have been cropped here.

GAPDH sample set 1

Figures 5D and 6B: normalization

V = Withdrawal+Vehicle; 3-MA = Withdrawal+3-MA; M = marker.

Chemiluminescence

GAPDH 36 kDa

GAPDH/set1_chemiluminescence.tif

							X	X	
	P321	P329	P322	P330	P323	P331	P324	P332	
**M**	**V**	**3-MA**	**V**	**3-MA**	**V**	**3-MA**	**V**	**3-MA**	**M**

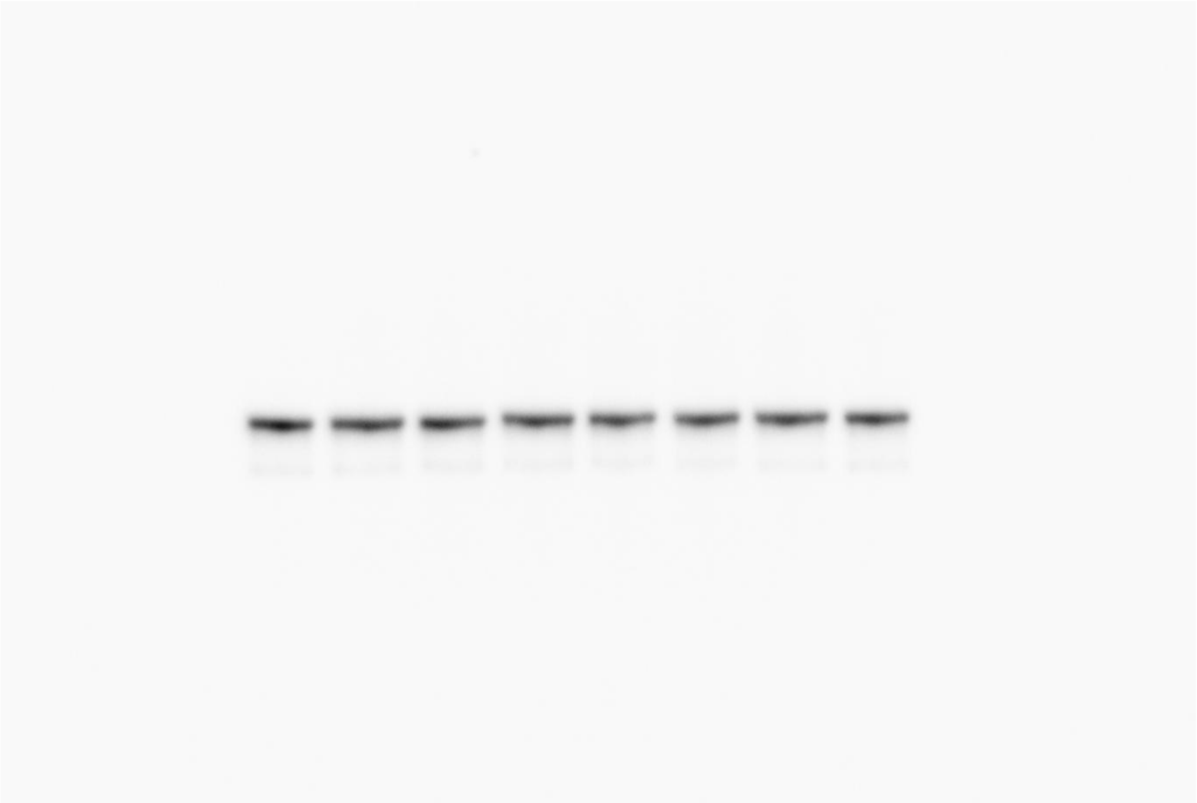

Representative panel: Figure 5B, sample lanes 1–6.

X = not displayed in the representative panel; all lanes included in the reported quantification.

White light

M 1 2 3 4 5 6 7 8 M

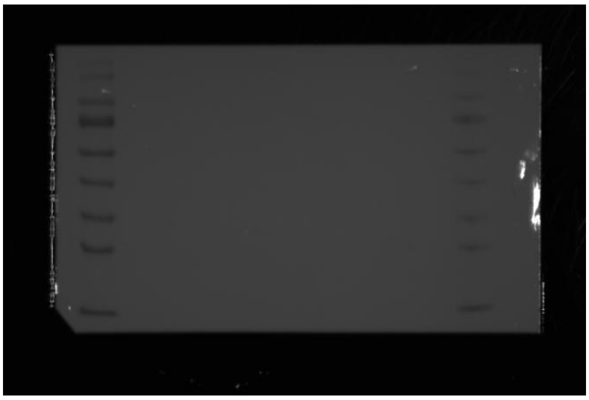

Merge (supplied overlay)

M 1 2 3 4 5 6 7 8 M

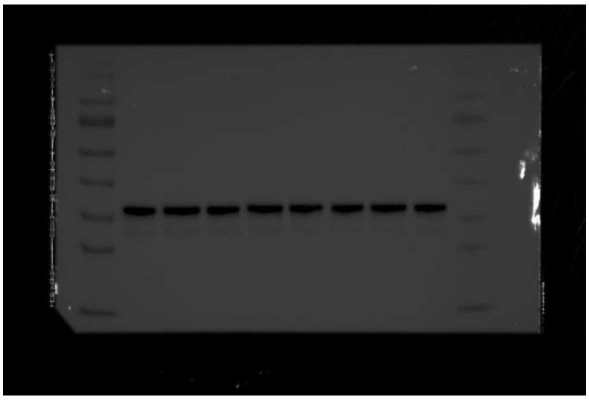

Lower-panel lane numbers refer to the mouse IDs above. No image fields have been cropped here.

GAPDH sample set 2

Figures 5D and 6B: normalization

V = Withdrawal+Vehicle; 3-MA = Withdrawal+3-MA; M = marker.

Chemiluminescence

GAPDH 36 kDa

GAPDH/set2_chemiluminescence.tif

							X	X	X	
	P325	P333	P326	P334	P327	P335	P328	P336	P337	
**M**	**V**	**3-MA**	**V**	**3-MA**	**V**	**3-MA**	**V**	**3-MA**	**3-MA**	**M**

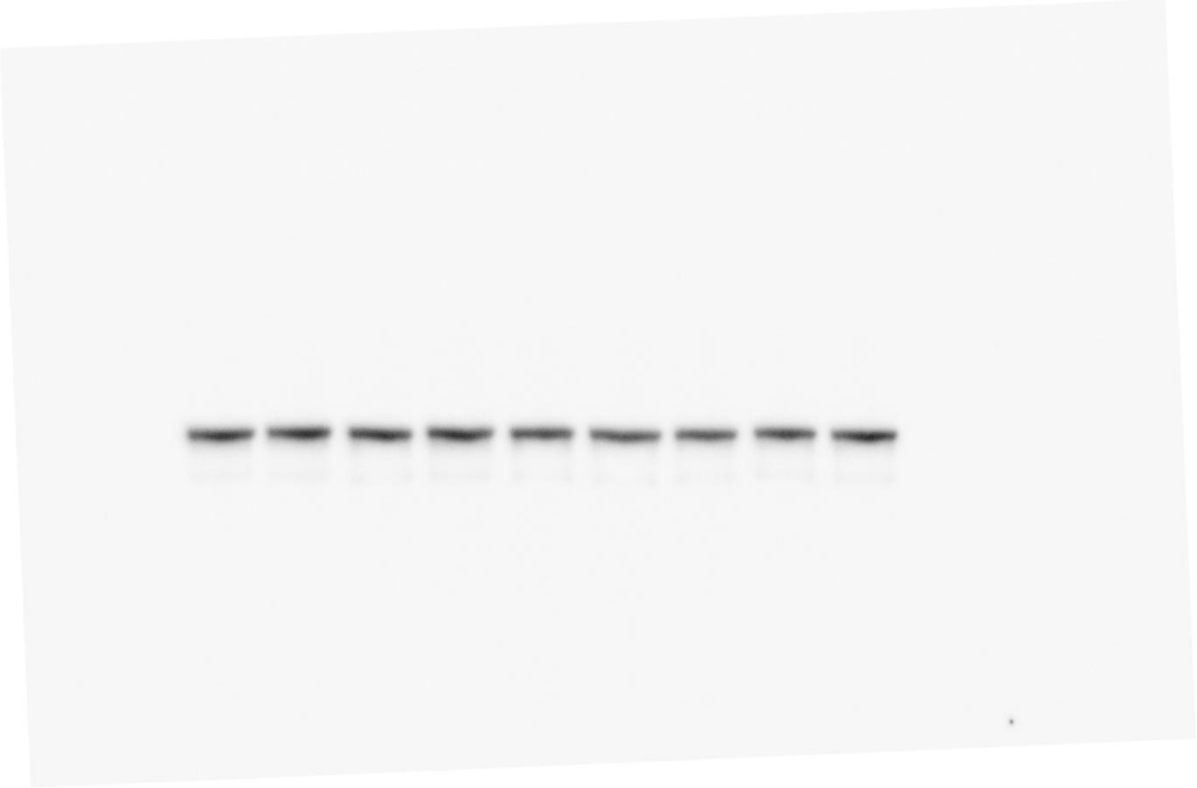

Representative panel: Figure 6A, sample lanes 1–6.

X = not displayed in the representative panel; all lanes included in the reported quantification.

White light

M 1 2 3 4 5 6 7 8 9 M

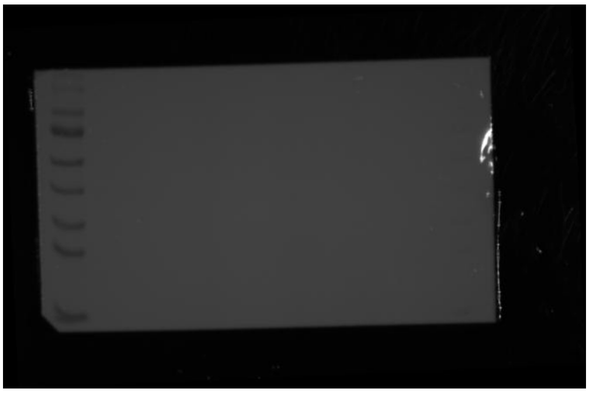

Merge (supplied overlay)

M 1 2 3 4 5 6 7 8 9 M

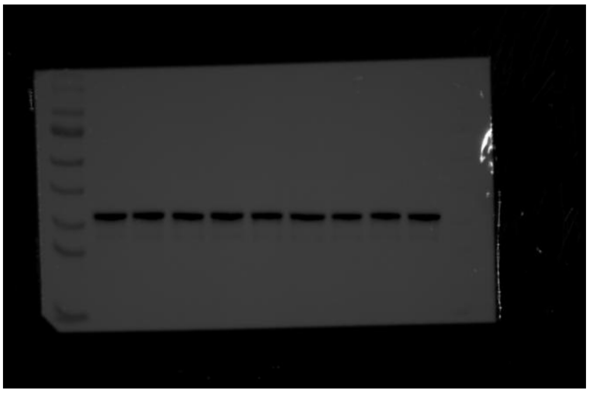

Lower-panel lane numbers refer to the mouse IDs above. No image fields have been cropped here.
